# New Network Polymer Electrolytes Based on Ionic Liquid and SiO_2_ Nanoparticles for Energy Storage Systems

**DOI:** 10.3390/membranes13060548

**Published:** 2023-05-24

**Authors:** Kyunsylu G. Khatmullina, Nikita A. Slesarenko, Alexander V. Chernyak, Guzaliya R. Baymuratova, Alena V. Yudina, Mikhail P. Berezin, Galiya Z. Tulibaeva, Anna A. Slesarenko, Alexander F. Shestakov, Olga V. Yarmolenko

**Affiliations:** 1 Federal Research Center of Problems of Chemical Physics and Medicinal Chemistry RAS, 142432 Chernogolovka, Russia; khatmullina_k@mail.ru (K.G.K.); wownik007@mail.ru (N.A.S.); sasha_cherniak@mail.ru (A.V.C.); guzalia.rb@yandex.ru (G.R.B.); gvinok@yandex.ru (A.V.Y.); berezin@icp.ac.ru (M.P.B.); tulibaeva@gmail.com (G.Z.T.); ansles@yandex.ru (A.A.S.); a.s@icp.ac.ru (A.F.S.); 2 Department of Chemistry and Electrochemical Energy, Institute of Energy Efficiency and Hydrogen Technologies (IEEHT), Moscow Power Engineering Institute, National Research University, 111250 Moscow, Russia; 3 Scientific Center in Chernogolovka of the Institute of Solid State Physics Named Yu.A. Osipyan RAS, 142432 Chernogolovka, Russia; 4 Faculty of Fundamental Physical and Chemical Engineering, M. V. Lomonosov Moscow State University, 119991 Moscow, Russia

**Keywords:** nanocomposite polymer gel electrolytes, SiO_2_ nanoparticles, NMR with PFG, self-diffusion coefficients, ionic conductivity, solid-state lithium battery, solvate shell, quantum-chemical modeling

## Abstract

Elementary processes of electro mass transfer in the nanocomposite polymer electrolyte system by pulse field gradient, spin echo NMR spectroscopy and the high-resolution NMR method together with electrochemical impedance spectroscopy are examined. The new nanocomposite polymer gel electrolytes consisted of polyethylene glycol diacrylate (PEGDA), salt LiBF_4_ and 1—ethyl—3—methylimidazolium tetrafluoroborate (EMIBF_4_) and SiO_2_ nanoparticles. Kinetics of the PEGDA matrix formation was studied by isothermal calorimetry. The flexible polymer–ionic liquid films were studied by IRFT spectroscopy, differential scanning calorimetry and temperature gravimetric analysis. The total conductivity in these systems was about 10^−4^ S cm^−1^ (−40 °C), 10^−3^ S cm^−1^ (25 °C) and 10^−2^ S cm^−1^ (100 °C). The method of quantum-chemical modeling of the interaction of SiO_2_ nanoparticles with ions showed the advantage of the mixed adsorption process, in which a negatively charged surface layer is formed from Li^+^ BF_4_^—^ ions on silicon dioxide particles and then from ions of the ionic liquid EMI^+^ BF_4_^−^. These electrolytes are promising for use both in lithium power sources and in supercapacitors. The paper shows preliminary tests of a lithium cell with an organic electrode based on a pentaazapentacene derivative for 110 charge–discharge cycles.

## 1. Introduction

In recent years, ionic liquids (ILs) have been increasingly used as components of polymer electrolytes for energy storage systems [1,2]. ILs have a number of advantages, such as low flammability, low vapor pressure and a wide thermal, chemical and electrochemical stability window [3].

Solid polymer electrolytes, based on various polymer matrices of poly(vinylidene fluoride) (PVdF), poly(vinylidene fluoride—co—hexafluoropropylene) (PVDF—HFP), poly(methyl methacrylate) (PMMA), poly(ethylene oxide) (PEO) and poly(acrylonitrile) (PAN), exhibit low ionic conductivity at room temperature; therefore, the search of suitable ILs for such systems is an actual problem [4,5].

A system with nanocomposites and ionic liquids is a new type of electrolyte system [6,7]. The nanocomposites contain particles such as Al_2_O_3_, SiO_2_, ZrO_2_, TiO_2_, SnO_2_, etc. [8,9,10,11,12,13].

He et al. [14] found that ILs are utilized as synthesis and dispersion media for nanoparticles as well as for surface functionalization. Ionic liquid and nanoparticle hybrid systems are governed by a combined effect of several intermolecular interactions between their constituents, for each interaction, including van der Waals, electrostatic, steric and hydrogen bonding. Various self-organized structures based on nanoparticles in ionic liquids are generated as a result of a balance of these intermolecular interactions. These structures, including nanoparticle-stabilized ionic-liquid-containing emulsions, possess properties of both ionic liquids and nanoparticles, which render them useful as novel materials, especially in electrochemical applications.

Unlike traditional salts, ionic liquids usually consist of large asymmetric polyatomic ions with an ionic radius above about 5 to 10 times that of monatomic ions such as Li^+^ or Na^+^. The large ionic size of the ion liquids increases the average distance between the charge cation and anion centers, reducing the electrostatic interaction strength. The ions or ion clusters are attracted to the nanoparticle surface by electrostatic forces [14]. The particles of metals have great attraction. The ionic liquid cations are attracted to the surface of a negatively charged nanoparticle to form a positive ion layer, and then counter ions form a second layer on the nanoparticle surface by electrostatic attraction.

In addition, there are nanoparticles that interact less with IL ions. One such example is colloidal fumed silica SiO_2_. Lithium salt additives are used to stabilize them, which is a requirement for the use of such systems in lithium-ion batteries. Thus, Nordström et al. [15] investigated the stability and interactions in dispersions of colloidal fumed silica SiO_2_ (Aerosil 200, Evonik Resource Efficiency GmbH, Antwerp, Belgium) and the ionic liquid 1—butyl—3—methylimidazolium tetraflouroborate (BMIBF_4_) as a function of the Li salt concentration (LiBF_4_). The increased stability with the addition of Li salt was found by Raman spectroscopy which is explained by the formation of a more stable solvation layer, where Li ions accumulate on the surface.

Electrolytes based on ionic liquids are mainly used in supercapacitors [16,17,18], where ionic liquid ions are charge carriers. There are also polymer electrolytes based on ILs for lithium current sources. Here, the competitive ion transport of lithium cations and IL cations has strong influences on the electrochemical processes [19,20,21,22,23].

The addition of different ionic liquids has various effects on the properties of Li^+^ ion conductive polymer electrolytes. In addition, the physical properties of ILs (in particular, viscosity and dielectric constant) have an important role in the structure design and conducting properties of polymer electrolytes. Low viscosity leads to an increase in the segmental mobility of the polymer chains. On the other hand, a high dielectric constant of the ionic liquid increases ion pair dissociation and, therefore, is the cause of the charge carrier concentration. Both of the above aspects contribute to the increase in polymer gel electrolyte ionic conductivity.

ILs with aprotic imidazolium cations are suitable for lithium batteries. In this study, 1—ethyl—3—methylimidazolium tetrafluoroborate (EMIBF_4_) was used, which shows the ion conductivity of 1.4 × 10^−2^ S cm^−1^ [24] and the electrochemical stability window of 4.0 V at room temperature [25].

This study deals with the synthesis and study of an electro mass transfer of new network polymer electrolytes based on an EMIBF_4_ ionic liquid with the introduction of various amounts of fumed silica SiO_2_ (Aerosil 380) nanoparticles (0, 2, 4, 6 wt.%). The SiO_2_ nanoparticles have a very small particle size of 7 nm with a narrow distribution and a very developed surface of 380 g/m^2^ due to porosity. On such a surface, lithium salt molecules can be adsorbed and dissociated into free ions [26,27,28,29].

We have previously studied polymer gel electrolytes based on polyethylene glycol diacrylate (PEGDA) as a polymer network, which showed good properties as a three-dimensional network with ethylene oxide units, which retain well a large amount of a liquid phase, consisting of both traditional aprotic solvents and ionic liquids such as EMIBF_4_ and 1—butyl—3—methylimidazolium tetrafluoroborate BMIBF_4_ [4,30]. The mechanism of ionic and molecular transport in new nanocomposite systems based on fumed SiO_2_ was investigated by the NMR method, electrochemical impedance spectroscopy and quantum-chemical modeling, which are the most informative methods for such complex systems.

## 2. Materials and Methods

### 2.1. Materials

LiBF_4_ (purity 98%) (lithium bis(trifluoromethanesulfonyl)imide) and LiTFSI (purity 99%, water ≤ 1%) were used as electrolyte salts; ethylene carbonate (EC, Aldrich, St. Louis, MO, USA, *T*_melt_ = 36 °C, purity ≥ 99%), 1,3—dioxolane (DOL, purity 99.8%) and dimethoxyethane (DME, purity 99%, water < 0.005%) were used as electrolyte solvents; 1—Ethyl—3—methylimidazolium tetrafluoroborate (EMIBF_4_, Aldrich, purity ≥ 98%) was used as ionic liquid. All chemical reagents and diluents were acquired from Sigma-Aldrich and used as received. Polyethylene glycol diacrylate (PEGDA, Aldrich, M_n_ = 700, *T*_melt_ = 12–17 °C) was used to obtain a three-dimensional network matrix for the polymer electrolyte. The radical polymerization initiator, benzoyl peroxide (PB, Aldrich), stored in water (30%) was recrystallized from chloroform followed by drying at 20 °C in air and then in a vacuum. SiO_2_ nanoparticles (average particle size 7 nm, Aerosil 380, Evonik Resource Efficiency GmbH, Rheinfelden, Germany) were used to fill the electrolyte polymer matrix. SiO_2_ nanoparticles had a hydrophilic surface, pH 3.6–4.3 (in 4% aqueous dispersion). Lithium foil (JSC “Lithium—element,” Saratov, Russia) 1 mm thick was the anode material.

**Figure 1:**
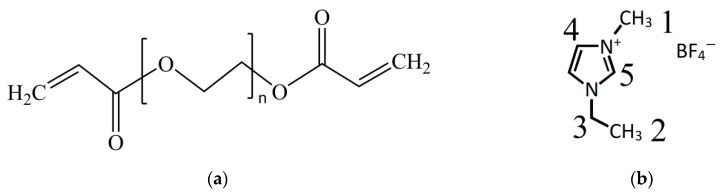
Structures of (**a**) polyethylene glycol diacrylate (PEGDA) and (**b**) ionic liquid EMIBF_4_, where digits (1–5) indicate the sites of the ^1^H and ^13^C atoms (for the description of the NMR spectra).

The structures of PEGDA and EMIBF_4_ are shown in Figure 1.

### 2.2. PEGDA Kinetics of Radical Polymerization

The kinetics of radical polymerization of PEGDA in the presence of the ionic liquid, salt LiBF_4_, EC, SiO_2_ nanopowder and benzoyl peroxide as an initiator was studied by isothermal calorimetry on a DAK—1—1 differential automatic calorimeter (EZAN, Chernogolovka, Russia) at 60 °C. The reaction mixture was placed into glass ampoules for calorimetric measurements and sealed.

### 2.3. Synthesis of Nanocomposite Polymer Electrolytes

Nanocomposite polymer electrolytes (NPEs) were synthesized by the radical polymerization of PEGDA in the presence of the radical initiator PB without an inert solvent.

The composition of the polymerizable mixture was as follows: PEGDA, LiBF_4_, EC, EMIBF_4_, SiO_2_ and 1 wt.% PB for the whole sample. The compositions of NPEs are listed in Table 1 in molar proportions of the components and in Table S1, ESI in mass percent, where the synthesis procedure in detail (ESI) is given.

To study samples by NMR method, the NPEs were synthesized in closed glass capillaries with a diameter of d = 4 mm, l = 50 mm. The capsules with NPEs were closed and placed in standard 5 mm ampoules for NMR examination.

**Figure 2:**
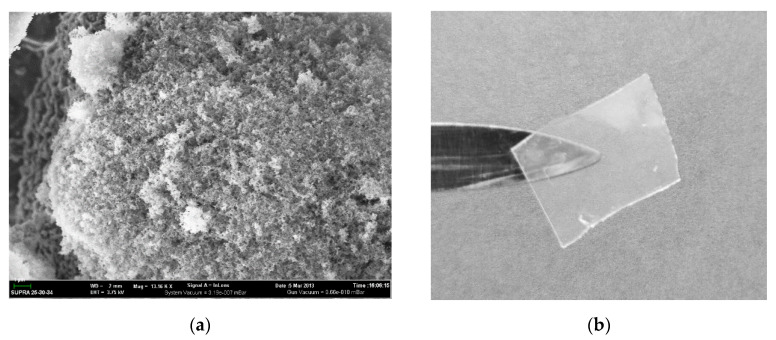
SEM image of the initial SiO_2_ powder (**a**) and an optical photo of the final NPE film (**b**).

The SEM image of the initial SiO_2_ powder is shown in Figure 2a.

An optical photo of the final nanocomposite electrolyte film is shown in Figure 2b. It can be seen from Figure 2b that the film is transparent.

### 2.4. Differential Scanning Calorimetry (DSC) Method

The glassy transition temperature in the temperature range from −150 to 50 °C and the homophase nature of the NPEs were determined from the differential scanning calorimetry (DSC) data obtained on a DSC 822e Mettler—Toledo instrument (Kutznacht an der Zürichsee, Switzerland) with the Star software at a scanning rate of 5 deg min^−1^.

### 2.5. Fourier Transform Infrared Spectroscopy (FTIR)

The FTIR spectra of thin-film electrolyte samples and initial components (PEGDA, IL and EC) were recorded on an IRTracer—100 FTIR spectrometer (Shimadzu, Germany) at room temperature in a wave number range of 400–4000 cm^−1^ with a spectral resolution of 2–4 cm^−1^.

### 2.6. Thermogravimetric Analysis (TGA) Method

The TGA data for the samples were obtained on a TGA/SDTA851 Mettler—Toledo instrument (China) in the temperature range from 30 to 150 °C at a heating rate of 5 deg min^−1^.

### 2.7. High-Resolution NMR

High-resolution spectra for ^1^H, ^7^Li, ^11^B, ^13^C and ^19^F were recorded on a Bruker Avance III 500 MHz NMR spectrometer. The measurements at frequencies of 500, 194, 160, 126 and 471 MHz for ^1^H, ^7^Li, ^11^B, ^13^C and ^19^F, respectively, were carried out at room temperature (22 ± 1 °C). The chemical shift scale was calibrated with the DMSO—d6 signal in the capillary as an external standard (2.50 ppm for ^1^H). The ^1^H, ^7^Li and ^19^F NMR spectra were obtained using the standard sequence π/2 pulses, FID. No signal accumulation was applied. To obtain the ^13^C NMR spectra, a standard sequence from the TopSpin (Bruker, Billerica, MA, USA) zgpg30 library was used. The sequence is an accumulation of signals from 30° pulses with the suppression of the ^1^H spin–spin interaction for the duration of all of the experimental times. The number of repetitions was ns = 512, and the delay between the repetition sequence was d1 = 1.0 s. For interpretation of ^1^H and ^13^C, two-dimensional ^13^C—^1^H HSQC correlation spectra were recorded (standard pulse sequence from the TopSpin library (Bruker)).

### 2.8. NMR with Pulsed Field Gradient

The NMR measurements on a Bruker Avance—III 400 MHz NMR spectrometer equipped with the diff60 gradient unit (the maximum field gradient amplitude was 30 T m^−1^) were carried out at the temperature 22 ± 1 °C. The NMR measurements of ^1^H (diffusion of solvent molecules EC and ionic liquid IL, EMI^−^), ^7^Li (diffusion of lithium cations) and ^19^F (diffusion of anions BF_4_^−^) were carried out with operating frequencies of 400, 155.5 and 376.5 MHz, respectively. The stimulated spin echo sequence was applied. The details of self-diffusion coefficient measurements are given in [31,32]. The experimental NMR parameters of pulse sequences were the following: π/2 pulse was 9 μs (^1^H), 9 μs (^7^Li) and 10 μs (^19^F); gradient pulse duration time δ was 1.0 (^1^H), 1.0 (^7^Li) and 3.0 (^19^F) ms; diffusion time was 19.7 (^1^H), 19.7 (^7^Li) and 49.0 (^19^F) ms; repetition time 3 s; and the diffusion 32 steps with maximum field gradient amplitude g were 3.5 (^1^H), 11.5 (^7^Li) and 4.0 (19F) T m^−1^. The strength of the gradient changed linearly. The measurement error of the self-diffusion coefficients was 5%. The temperature dependences of the diffusion coefficients were measured at the temperature range from 22 to 60 °C.

### 2.9. Electrochemical Methods

To measure the conductivity of NPE film samples by the electrochemical impedance method in symmetrical stainless steel cells (SS)//SS with an area equal to 0.2 cm^2^, a Z—2000 impedance meter (Elins, Chernogolovka, Russia) was used in the frequency range from 1 Hz to 2 MHz with a signal amplitude of 10 mV. The cell impedance was detected in the temperature range from −40 to 100 °C. Four measurements were carried out for each sample. The measurement error was not higher than 2%.

Symmetrical cells with Li metal and LiOTAP organic cathodes were assembled in coin-type cells CR2032. To measure the resistance of the boundary of NPE/electrode by the electrochemical impedance method in symmetrical cells Li/Li and LiOTAP/LiOTAP, a Z—2000 impedance meter was used analogically.

The electrochemical performance of the Li//LiOTAP batteries was evaluated using a BTS—5 V 10 mA battery analyzer (Neware Technology Ltd., Shenzhen, China) by performing charge/discharge cycling at current rates C/2 in a range of 0.7–3.5 V. LiOTAP was synthesized and characterized in [33]. The electrochemical performance of LiOTAP was evaluated in coin-type lithium batteries. The cathode composition comprised 45 wt.% of LiOTAP, 50 wt.% of conductive carbon black (Timical Super C65) and 5 wt.% of PVDF polymer binder (Kynar Flex HSV 900, Arkema, Colombes, France). The procedure for assembling cells with a polymer electrolyte differed from that mentioned in [33] in that an NPE film was placed instead of a separator with a liquid electrolyte.

### 2.10. Quantum-Chemical Modeling

The structure of complexes of different ions with solvent molecules and SiO_2_ was studied using the nonempirical Perdew–Burke–Erzernhof (PBE) exchange-correlation functional [34] using the extended basis H [5s1p/2s1p], C, N, O, F, S [5s5p2d/3s3p2d] and Li [4s1p/2s1p] for valence electrons and SBK pseudopotential [35]. The geometry of larger systems containing a counterion and additional solvent molecules was optimized using the effective Hamiltonian method [36] taking into account the van der Waals interaction. The Priroda package [37] was used for all the calculations carried out at the Joint Supercomputer Center of the Russian Academy of Sciences.

## 3. Results and Discussion

### 3.1. PEGDA Radical Polymerization Kinetics

**Figure 3:**
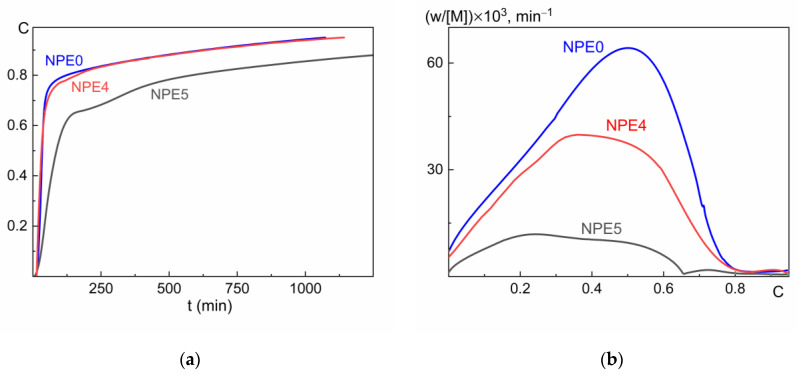
Dependence of the conversion (*C*) (**a**) and the reduced polymerization rate (w/[M]) of the NPE samples (**b**) on time (*t)* in the presence of air oxygen. *T* = 60 °C.

Figure 3 shows that SiO_2_ nanoparticles had an inhibitory effect on polymerization. A clear dependence of polymerization on the stability of SiO_2_ with a particularly strong influence of the inhibitory effect on the maximum rate was observed.

The kinetic curves (Figure 3a) followed that the main part of the polymerization of the studied compositions takes about 3 h. To accelerate the synthesis time of finished electrolyte films, it is necessary to carry out polymerization in a stepwise mode (60, 70 and 80 °C). This is because an increase in temperature by 10° leads to an increase in the polymerization rate by several times. Therefore, the post-polymerization time reduced, and the polymer electrolytes with a maximum conversion were obtained.

### 3.2. DSC of NPEs

**Figure 4:**
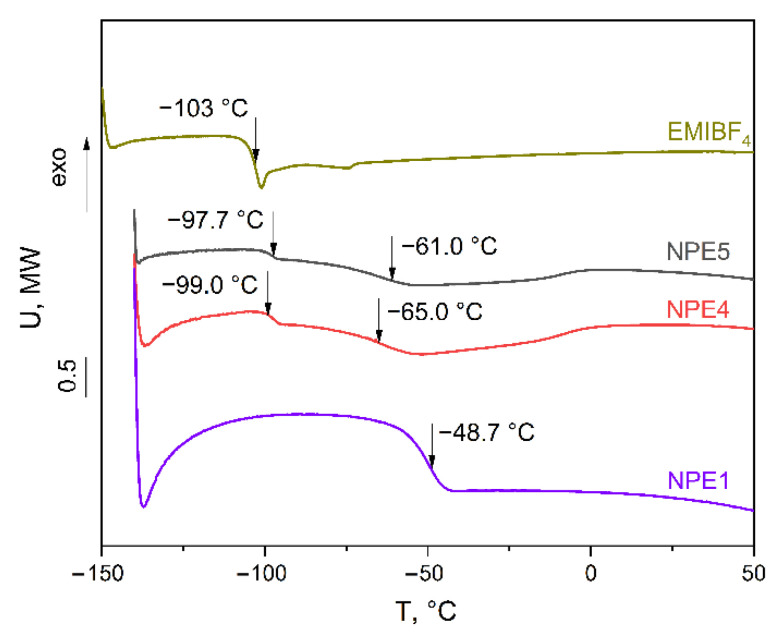
DSC curves for NPE1, 4–5 compositions and ionic liquid EMIBF_4_.

All compositions of NPEs and initial ionic liquid were studied by DSC. The ionic liquid had only one phase transition; the glass transition temperature T_g_ = −103 °C. All NPE samples had two T_g_ values that characterized EMIBF_4_ and PEGDA. The results are presented in Table 2. Figure 4 shows the DSC diagrams of EMIBF_4_, NPE1, NPE4 and NPE5 as an example.

### 3.3. FTIR Analysis of NPEs

The peak of the carbonyl group of PEGDA at 1721 cm^−1^ shifts to a range of 1733 cm^−1^, which is caused by the three-dimensional crosslinking of diacrylate in a medium of a large amount of the liquid phase as shown by the quantum-chemical modeling of PEGDA crosslinking (Figure S1, ESI) and confirmed experimentally in [38].

The peak of the carbonyl group of ethylene carbonate in the composition of PEGDA—LiBF_4_—3EC undergoes a strong shift. This is due to the formation of a solvate shell of the lithium cation by EC molecules. The theoretical IR spectra of the LiBF_4_—3EC solvate showed this effect (Figure S2, ESI).

**Figure 5:**
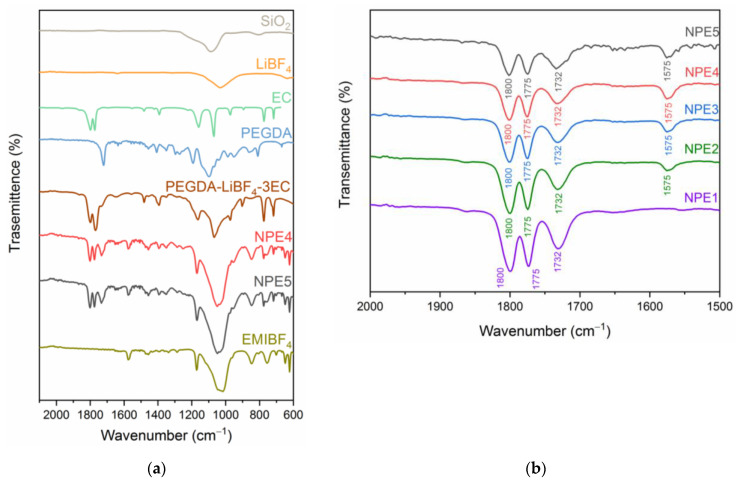
FTIR spectra for the NPE4 and NPE6 compositions compared with the EC (liquid, 40 °C), polymer PEGDA, ionic liquid EMIBF_4_, nanoparticles SiO_2_, salt LiBF_4_ and polymer electrolyte (PEGDA—LiBF_4_—3EC) (**a**) and FTIR spectra for all NPE1—5 (**b**).

The peaks of the carbonyl group of EC upon the addition of SiO_2_ nanoparticles return to their original position (Figure 5). Most likely, they came out of the EC solvate environment. As can be seen in Figure S3, ESI showed an enlarged spectrum of the carbonyl group.

### 3.4. TGA of NPEs

**Figure 6:**
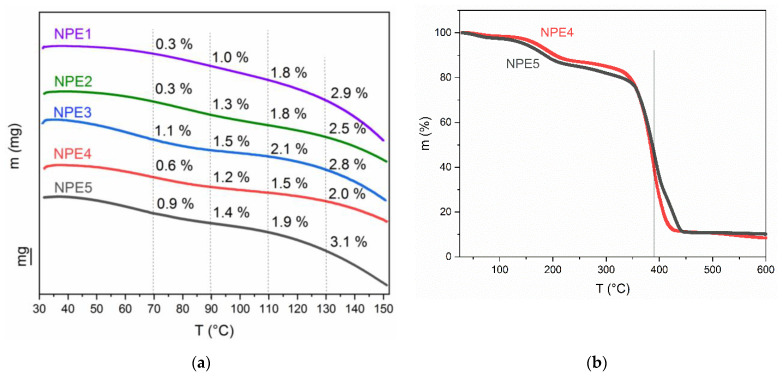
TGA diagrams of all five NPE compositions ranging from room temperature to 150 °C (**a**) and NPE4 and NPE5 compositions up to 600 °C (**b**).

The TGA dependences of all NPE compositions are shown in Figure 6.

It is seen from Figure 6a that the polymer electrolytes are stable up to 100 °C. Instrumental inaccuracy is the mass loss of up to 1%. A slight weight loss may indicate a loss of moisture. Moisture during preparation for the study could get into the sample. The end of the first stage of the TGA diagram at 100 °C confirmed this. In addition, when examining samples by IR spectroscopy before and after heating up to 150 °C, it was shown that the main peaks of all components did not change (Figure S4, ESI).

Figure 6b shows the TGA diagrams up to 600 °C of the NPE4 and NPE5 compositions with maximum conductivity.

Figure 6b shows the multistage character of sample weight loss. This explains the gradual loss of each component. The loss of ethylene carbonate (bp = 248 °C) occurs first. The ionic liquid, apparently, decomposes together with the polymer matrix at 390 °C. Silicon dioxide remains in the residue. Under extreme conditions, it is possible that SiO_2_ will insulate between the electrodes (if they are stable up to these temperatures).

### 3.5. High-Resolution NMR

**Figure 7:**
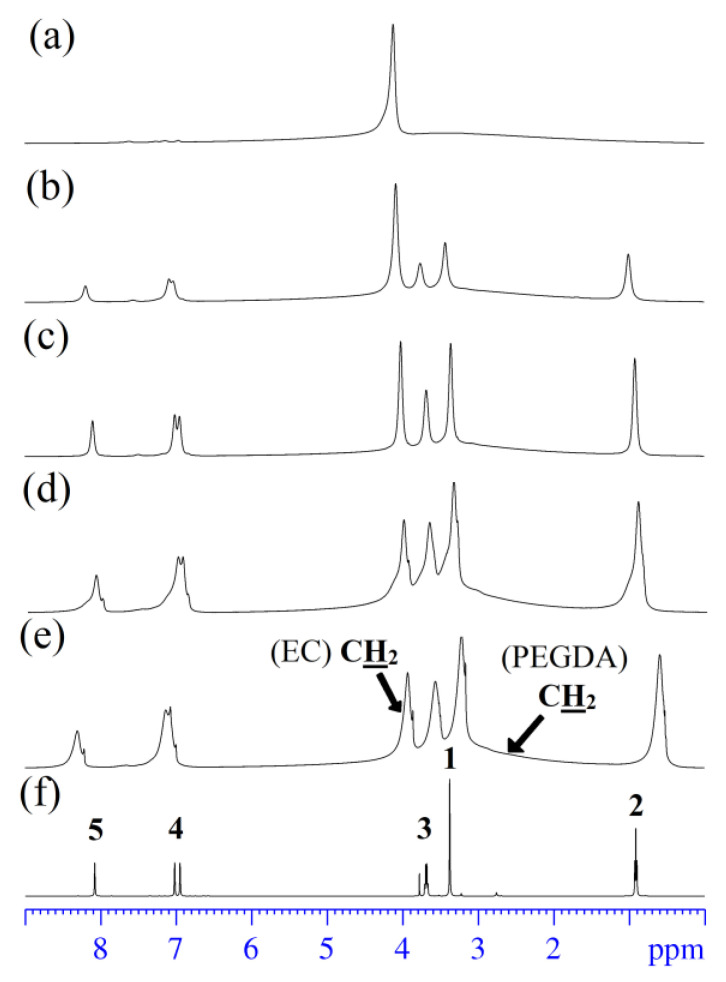
^1^H NMR spectra of the electrolytes (**a**) NPE1, (**b**) NPE2, (**c**) NPE3, (**d**) NPE4, (**e**) NPE5 and (**f**) ionic liquid EMIBF_4_ (atomic numeration in (**f**) is indicated taking into account Figure 1b).

**Figure 8:**
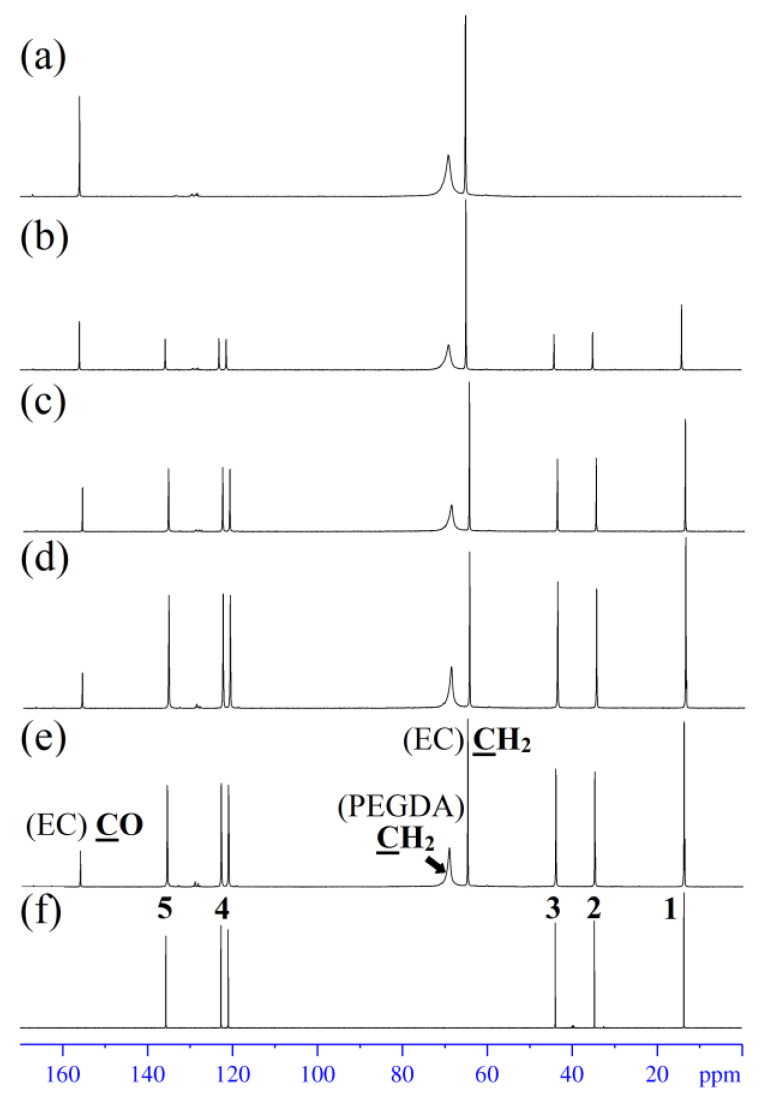
^13^ C NMR spectra of the electrolytes (**a**) NPE1, (**b**) NPE2, (**c**) NPE3, (**d**) NPE4, (**e**) NPE5 and (**f**) ionic liquid EMIBF_4_ (atomic numeration in (**f**) is indicated taking into account Figure 1b).

The ^1^H and ^13^C NMR spectra to check the purity and confirm the NPE compositions were recorded. The ^1^H and ^13^C NMR spectra for all NPEs compared to the ionic liquid are shown in Figure 7 and Figure 8, respectively. The ^1^H and ^13^C spectra differ in integral signal intensities due to different molar ratios of EMIBF_4_ to solvent. The ^7^Li, ^19^F and ^11^B NMR spectra were also obtained (Figures S5–S7, ESI).

Figure 7 shows that the signals in the ^1^H NMR spectra of the polymer electrolytes are significantly broader than those in the pure EMIBF_4_. The signal of ethylene carbonate is also broadened (~4 ppm). The signal broadening is caused by the formation of a branched network polymer structure formed by PEGDA, which considerably impedes the chaotic motion of EMIBF_4_ and EC [39]. The ^1^H NMR spectrum of the electrolyte exhibits a very broad signal from —O—CH_2_—CH_2_—O— of the polymer matrix with a maximum at ~3 ppm. This signal in the ^13^C—^1^H HSQC spectrum correlates with the ^13^C signal at 69.2 ppm in the ^13^C NMR spectrum (Figure S8, ESI).

**Figure 9:**
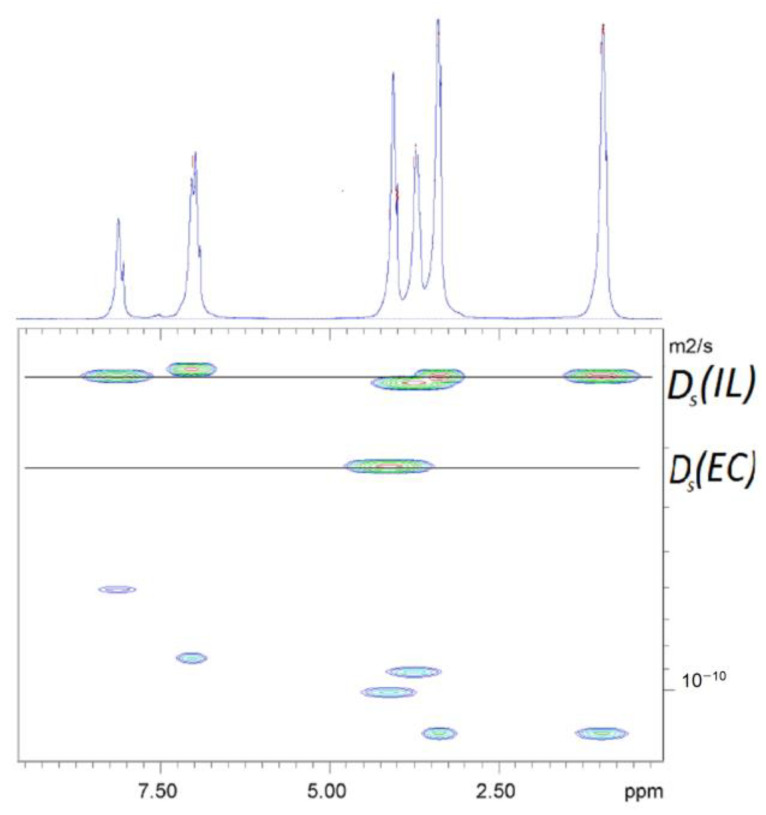
^1^H 2D DOSY NMR spectra of EMIBF_4_ (IL) and EC for NPE4.

A 2D DOSY spectrum to confirm signal assignment in the proton spectrum was also recorded (Figure 9).

A spectrum DOSY signal in the spectrum of a mixture of molecules depending on the diffusion coefficients (Y-axis, Ds) allows them to separate. The EC solvent molecule is smaller than the IL and therefore more mobile. Thus, the signals from the less mobile IL (Ds(IL)) and the signal from the EC (Ds(EC)) from Figure 9 are easy to separate.

### 3.6. Self-Diffusion Coefficients (SDCs) According to the PFG NMR Data

The SDCs on ^1^H, ^7^Li and ^19^F for all the NPE compositions were measured by NMR with PGF. The diffusion decays on all nuclei of all compositions were exponential (Figure S9, ESI). The measurements of the self-diffusion coefficients *D_s_* on ^1^H make it possible to determine the mobility of EMIBF_4_ and EC (the analysis of diffusion decays of the signals from the ionic liquid or ethylene carbonate solvent allows one to estimate their mobilities separately). The *D_s_* on ^7^Li corresponds to the mobility of lithium cations, and that on ^19^F corresponds to the mobility of the BF_4_^−^ anion.

#### 3.6.1. Self-Diffusion Coefficients on ^1^H Nucleus

The results of measuring *D_s_* for the NPE1—5 compositions are given in Table 3. The *D_s_* values for pure ionic liquid EMIBF_4_ are presented for comparison.

Table 3 shows that the mobility of EC and IL molecules increases with an increase in the fraction of IL in the polymer electrolyte.

The *D_s_* of EC increases by more than six times with an increase in the mass content of IL from 0 to 6% (compositions NPE1—4). In this case, the *D_s_* of IL increases by four times. This is probably related to the “loosening” of the electrolyte polymer network upon the introduction of IL molecules. In this case, the *D_s_* of pure IL is more than an order of magnitude higher than the *D_s_* of IL in the polymer electrolyte (NPE 2). An increase in the content of the SiO_2_ additive at the same mass content of IL (NPE4 and NPE5) does not lead to a significant change in the mobility of the NPE components.

**Figure 10:**
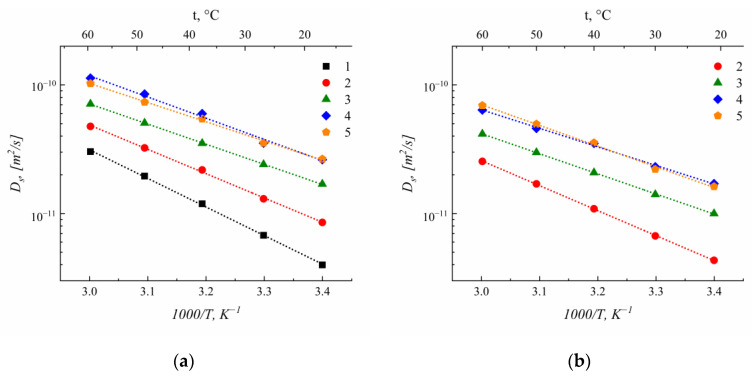
Temperature dependences of the diffusion coefficients on ^1^H nucleus of (**a**) EC and (**b**) EMIBF_4_.

The temperature dependences of the self-diffusion coefficients *D_s_* on ^1^H for EMIBF_4_ and EC molecules were measured in the range from 22 to 60 °C (Figure 10). Self-diffusion coefficient temperature *D_s_(T)* dependences are approximated by the Arrhenius equitation:

*D*_*s*_(*T*) = *D*_0_ ∙ exp(−*E*_*a*_/*RT*), (1)

where *D*_0_ is the temperature independent value, *R* is a gas constant, and *T* is the absolute temperature. *E_a_* is self-diffusion activation energy.

The dependences are Arrhenius. The activation energies of diffusion were calculated (Table 3). It is shown that an increase in the IL content in the composition of the polymer electrolyte leads to a decrease in the activation energy of the diffusion of EC and IL molecules up to ~40 to ~30 kJ mol^−1^. The activation energy of the diffusion of the pure ionic liquid was 21 kJ mol^−1^.

#### 3.6.2. Self-Diffusion Coefficients on ^7^Li Nucleus

The results of measuring *D_s_* (^7^Li) for the NPE1—5 compositions are given in Table 4.

An increase in the SDC for Li^+^ cations with an increase in the IL content in the polymer electrolyte, as in the case of the molecular mobility of the IL and EC components, was observed. The D_s_ of lithium cations increases by more than an order of magnitude (with an increase in the mass content of IL from 0 to 6 wt.%).

An increase in the addition of SiO_2_ from 2 to 6 wt.% (transition from composition 4 to composition 5) leads to a slight increase in the D_s_ of lithium cations. Note that the SDC (Li^+^) is ten times lower than the SDC of EMIBF_4_ despite the small size of the lithium cation compared to the IL molecule.

**Figure 11:**
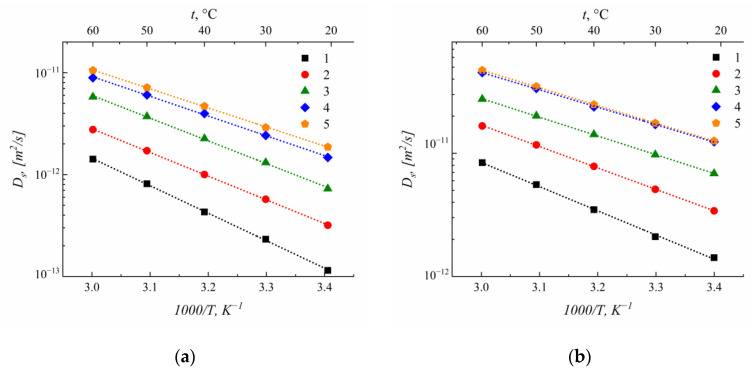
Temperature dependences of the diffusion coefficients on (**a**) ^7^Li and (**b**) ^19^F nuclei.

The temperature dependences of the self-diffusion coefficients *D_s_* on ^7^Li were measured in the range from 22 to 60 °C (Figure 11a). 

The dependences are Arrhenius. The activation energies of diffusion were calculated (Table 4). Both *E_a_* values for ^7^Li and ^19^F nuclei increase upon passing from the NPE5 to NPE1 composition (with a decrease in the IL content in the composite). It is shown that an increase in the IL content in the composition of the polymer electrolyte leads to a decrease in the activation energy of the diffusion of Li^+^ from ~50 to ~35 kJ mol^−1^.

#### 3.6.3. Self-Diffusion Coefficients on ^19^F Nucleus

The ^19^F NMR spectrum (Figure S7, ESI) shows one singlet, which is a superposition of the signals from the lithium salt anion BF_4_^−^ and the second BF_4_^−^ in the EMIBF_4_ ionic liquid. This was caused by the rapid chemical exchange of BF_4_^−^. Thus, the SDCs measured on the ^19^F nuclei are the weighted average of the mobility of the BF_4_^−^ and BF_4_^−^ anions in EMIBF_4_.

Table 4 shows that the mobility of BF_4_^−^ increases by an order of magnitude with an increase in the mass content of IL from 0 to 6% (compositions NPE1—4). An increase in the content of the SiO_2_ additive at the same mass content of IL (compositions 4 and 5) does not lead to a significant change in the mobility of BF_4_^−^ cations in the NPEs.

The temperature dependences of the self-diffusion coefficients *D_s_* on ^19^F were measured in the range from 22 to 60 °C (Figure 11b). The dependences are Arrhenius. The diffusion activation energies calculated by Formula (1) are presented in Table 4. It is shown that an increase in the IL content in the composition of the polymer electrolyte leads to a decrease in the activation energy of the diffusion of BF_4_^−^ from ~37 to ~27 kJ mol^−1^.

Thus, IL molecules contribute to an increase in the mobility of all components in the polymer matrix. The SDCs of Li^+^ and BF_4_^−^ increase by an order of magnitude with an increase in the mass content of IL from 0 to 6% (compositions NPE1—4).

The Li^+^ cation has the lowest diffusion coefficient D_s_. According to the obtained data, D_s_ (EMI^+^) > Ds (BF_4_^−^) >> Ds(Li^+^). Increasing the addition of SiO_2_ from 2 to 6 wt.% (transition from composition 4 to composition 5) leads to a slight increase in the D_s_ of lithium cations.

### 3.7. NPE Conductivity

**Figure 12:**
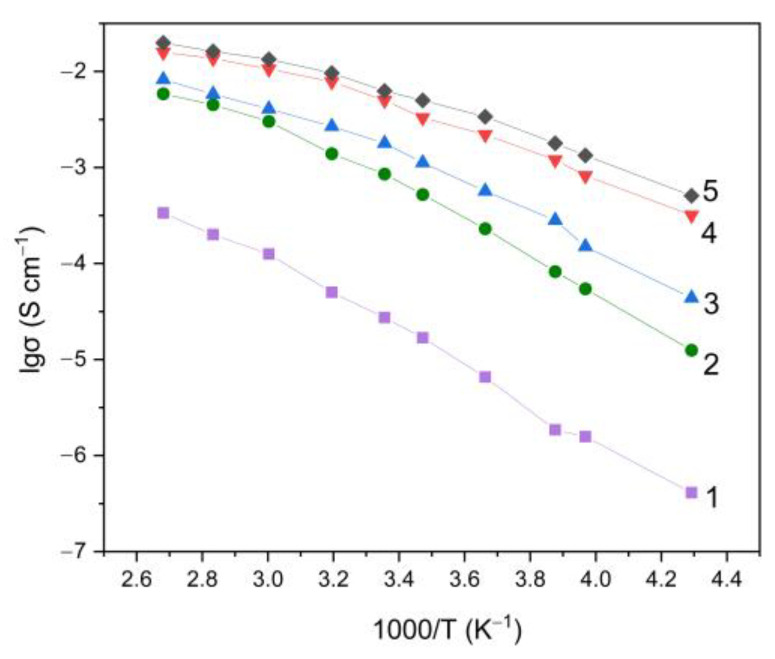
Temperature dependences of the NPE conductivity.

The conductivity of the obtained NPE samples was measured by the electrochemical impedance method in symmetrical SS//SS cells in the temperature range from −40 °C to 100 °C. The typical Nyquist plots of the cells are shown in Figure S10, ESI. The measurement results are given in Table S2, ESI and in Figure 12.

The Arrhenius temperature dependence of the conductivity for all compositions (Figure 12) had a break in the temperature range from 15 to 25 °C, and, hence, the effective activation energy of conductivity was calculated in two ranges (Table 5). The activation energies of conductivity and diffusion are compared. For the NPE1 composition, the activation energy of conductivity is noticeably lower than the activation energy of ^7^Li diffusion and comparable with the activation energies of ^19^F diffusion. With an increase in ionic liquid content, there is the same tendency of activation energies, but *E_a_* values for conductivity decrease most strongly, 2.5 times.

It is seen from Figure 12 and Table 5 that the composition with 6 mol IL (NPE4 and NPE5) has the highest conductivity and the most effective activation energy among all thin-film electrolytes. However, these values are higher for the NPE5 composition (with 6 wt.% SiO_2_). This indicates the positive contribution of SiO_2_ nanoparticles.

### 3.8. Electrochemical Study of NPEs in Li//LiOTAP Cells

**Scheme 1:**
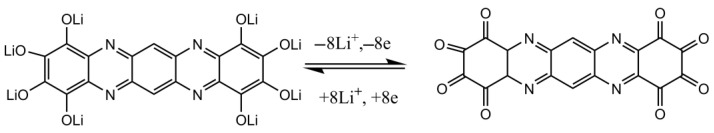
Electrochemical reduction and oxidation of LiOTAP.

In this work, battery prototypes with a cathode based on the lithium salt of the tetraazapentacene derivative LiOTAP were assembled. The electrochemical reduction and oxidation of LiOTAP are shown in Scheme 1. Each molecule of LiOTAP can undergo eight-electron oxidation also releasing eight Li^+^ cations, which corresponds to the theoretical specific capacity of 468 mA h g^−1^.

**Figure 13:**
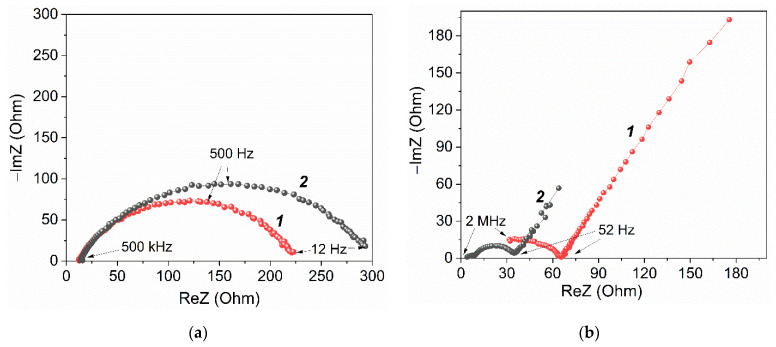
Nyquist plots of (**a**) Li//Li (*1*—NPE4; *2*—NPE5) and (**b**) LiOTAP/NPE4/LiOTAP cells (*1*—without, *2*—with liquid electrolyte).

First, the compatibility of NPE3—5 with Li-anode and LiOTAP-cathode materials was investigated by the electrochemical impedance method. The method of “liquid-phase therapy” for LiOTAP//LiOTAP cells was applied. The liquid electrolyte 1M LiTFSI in DOL/DME (1:1 vol.) was used similar to [39]. The results are shown in Figure 13.

**Figure 14:**
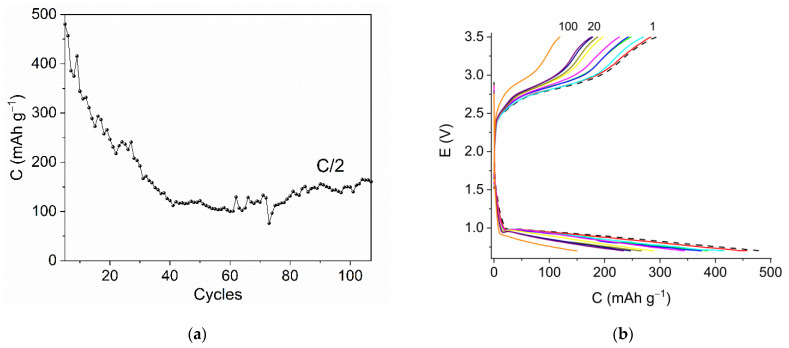
Dependence of the discharge capacity on the cycle number (**a**) for the Li/NPE3/LiOTAP cells at the C/2 current rate in a voltage range of 0.7–3.5 V. The charge–discharge profiles (**b**) of Li/NPE3/LiOTAP cells at C/2 current for the 1–20, 100 cycle numbers.

Figure 13 shows that both electrode materials are compatible with the obtained NPEs. The calculation of the equivalent circuit parameters for the Li/NPE4/Li cell is shown in detail in Figure S11, ESI. Treatment of the surface of the porous LiOTAP cathode with a liquid electrolyte reduced the resistance at the interface. Thus, Li/NPE3/LiOTAP cells with cathode treatment were chosen for life tests. The test results for 110 charge–discharge cycles are shown in Figure 14a. Figure 14b shows the charge–discharge profiles of Li/NPE3/LiOTAP cells at C/2 current for the 1–20, 100 cycle numbers. Figure S12 (ESI) shows the Coulomb efficiency (*CE*) during the cycling of this cell.

Figure 14 shows that the LiOTAP organic cathode material loses its capacity in the first cycles but then stabilizes at 150 mAh g^−1^. This substance belongs to the class of “small molecules” and is able to dissolve in the process of charge–discharge. The effect of LiOTAP dissolution in a liquid electrolyte was observed in [33]. However, with the use of a polymer electrolyte, no LiOTAP dissolution was observed in this work. This was confirmed by IR spectroscopy in the study of NPE4 samples before and after charge–discharges cycling. The opening of the cell after cycling was made in an Ar glove box. The lithium anode remained a shiny metal. No characteristic peaks of the IR spectra of the LiOTAP material in the film composition were observed (Figure S13, ESI).

A significant excess of the Coulomb efficiency above 100% can be seen in Figure S12, ESI. Obviously, this effect is due to SiO_2_ nanoparticles, since in their absence, the reversible operation of the Li/EMIBF_4_/LiOTAP cell was fixed, although short-lived (Figure S14, ESI). We attribute this effect to the reduction reaction of the EMI^+^ cation on the surface of the SiO_2_ nanoparticle. Quantum-chemical calculations show a significant binding energy (49.4 kcal mol^−1^ or 2.14 eV) of two radicals formed during the reduction of EMI^+^ (Figure S15a, ESI). An excess amount of discharge capacity over charging capacity (Figure 14b) occurs at low potentials of 0.7 V. Apparently, here, the activity of an ionic liquid with a carbon material (50 wt.% of cathode) similar to supercapacitors or a dual graphite battery manifests itself [40,41,42,43,44]. The Coulomb efficiency would be 100% if EMIBF_4_ remained unchanged.

We suppose that during a long cycle (24 h), the next reaction has time to occur. An electron from the carbon material is transferred to the imidazolium cation, and the resulting di-radical (Figure S15a, ESI) binds to the surface of the SiO_2_ nanoparticle (Figure S15b, ESI). This radical then adds the next radical but with a significant gain in energy. This mechanism was confirmed by the presence of changes in the IR spectra of the polymer electrolyte before and after charge–discharge cycling in the battery (Figure S16, ESI).

The discharge capacity in the first cycle is close to the theoretical one (8e^−^ LiOTAP redox transition) (Figure 14). Subsequently, it decreases to 150 mAh g^−1^, which corresponds to a two-electron transition. Since we consider the active participation of the EMI^+^ cation in these processes, it can be assumed that during the discharge, a partial insertion of the bulky EMI^+^ cation instead of Li^+^ occurs. These bulky cations shield the redox active groups of LiOTAP, and the capacitance efficiency drops in the first 40 charge–discharge cycles.

### 3.9. Quantum-Chemical Modeling

**Figure 15:**
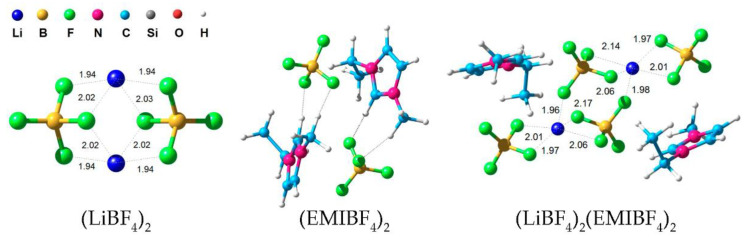
Various cluster models of LiBF_4_ salt and EMIBF_4_ ionic liquid.

Using quantum-chemical calculations by means of the density functional theory, models of the LiBF_4_ salt and the EMIBF_4_ ionic liquid were constructed in the form of an associate of two ion pairs, as well as a (LiBF_4_)_2_(EMIBF_4_)_2_ cluster containing both types of associates (Figure 15).

**Figure 16:**
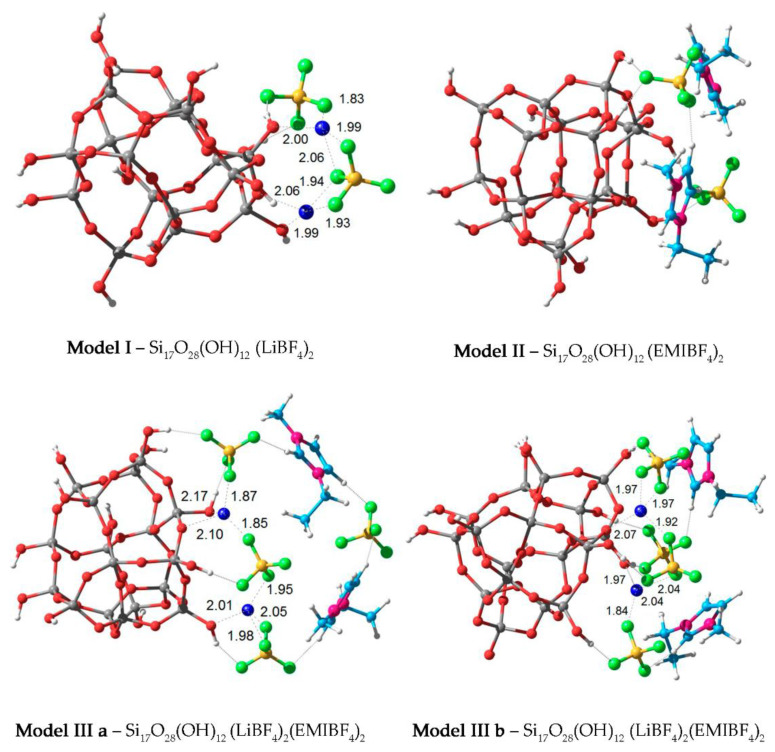
Various models of adsorption of ionic complexes on the surface of SiO_2_ nanoparticles.

As a model of silicon dioxide nanoparticles, we considered a cluster with a core of 17 bound SiO_2_ molecules and a hydrated surface as a result of the addition of 6 H_2_O molecules (Figure 16).

The interaction of the Si_17_O_28_(OH)_12_ cluster and the LiBF_4_ salt (**Model I**) results in the formation of additional Li–O coordination bonds and OH...FBF_3_^−^ hydrogen bonds, which leads to a decrease in the energy of the system by 23.5 kcal mol^−1^. During the interaction of a silicon dioxide nanoparticle and an ionic liquid, only strong hydrogen bonds are formed (**Model II**), which is also accompanied by an energy gain of 33.6 kcal mol^−1^.

**Model IIIa** and **Model IIIb** describing the interaction of a nanoparticle with a mixed (LiBF_4_)_2_(EMIBF_4_)_2_ cluster differ in the number of BF_4_^–^ ions directly in contact with the SiO_2_ surface. The process of adsorption of the (LiBF_4_)_2_(EMIBF_4_)_2_ cluster on the surface of the SiO_2_ nanoparticle proceeds with an energy gain of 17.4 and 33.9 kcal mol^−1^ with the formation of **Model IIIa** and **Model IIIb** structures, respectively.

At the same time, the incorporation of the (EMIBF_4_)_2_ cluster into the surface layer of ions for **Model I** leads to a greater gain in energy (34.6 and 51.1 kcal mol^−1^, respectively). This indicates the advantage of the mixed adsorption process, which results in the formation of a negatively charged surface layer of Li^+^ and BF_4_^–^ ions.

This conclusion is consistent with the results of experimental work [15] on the study of the interaction of SiO_2_ (Aerosil 200), LiBF_4_ and BMIBF_4_ nanoparticles using Raman spectroscopy. In the case of our system with SiO_2_ nanoparticles (Aerosil 380), due to their highly porous structure, the Raman spectra turned out to be not informative.

## 4. Conclusions

New nanocomposite polymer gel electrolytes based on polyethylene glycol diacrylate (PEGDA), LiBF_4_ salt and 1—ethyl—3—methylimidazolium tetrafluoroborate (EMIBF_4_) and SiO_2_ nanoparticles (Aerosil 380) were synthesized and studied. The SiO_2_ nanoparticles with a highly porous surface are unique in their nature of interaction with ions of lithium salt ions and ionic liquid. In this work, the electro mass transfer in such complex systems was investigated. The NMR method of high resolution and with a pulsed field gradient was used. Together with the electrochemical impedance data, information was obtained on the high conductivity of these systems. The total electrical conductivity was about 10^−4^ S cm^−1^ (−40 °C), 10^−3^ S cm^−1^ (25 °C) and 10^−2^ S cm^−1^ (100 °C). Based on the obtained data, quantum-chemical modeling of the adsorption of ionic complexes on the surface of a SiO_2_ nanoparticle was carried out. It was shown that the most energy-efficient process is mixed adsorption, which forms a negatively charged surface layer of Li^+^ BF_4_^−^ ions and then EMI^+^ BF_4_^−^ ions. This is also confirmed by the results of NMR with a pulsed field gradient.

These electrolytes are promising for use both in lithium power sources and in supercapacitors. This paper shows the preliminary tests of a lithium cell with an organic electrode based on a pentaazapentacene derivative for 110 charge–discharge cycles.

## Figures and Tables

**Table 1:** Compositions of the nanocomposite polymer gel electrolytes.

No.	PEGDA, mol	LiBF_4_, mol	EMIBF_4_, mol	EC, mol	SiO_2,_ wt.%	PB, wt.%
NPE0	1	1	6	3	0	1
NPE1	1	1	0	3	2	1
NPE2	1	1	2	3	2	1
NPE3	1	1	4	3	2	1
NPE4	1	1	6	3	2	1
NPE5	1	1	6	3	6	1

**Table 2:** Glass transition temperatures (°C) of the NPEs (according to the DSC data).

Sample	*T*_01_, °C	*T_g_*_1_, °C	∆*T*_1_, °C	*T_g_*_2_, °C	∆*T*_2_, °C
EMIBF_4_	−	−	−	−103.0	3.0
NPE0	−78.0	−61.5	32.9	−102.3	4.3
NPE1	−54.5	−48.7	9.57	−	−
NPE2	−64.5	−56.2	12.5	−98.2	4.2
NPE3	−68.9	−57.9	18.5	−98.3	4.0
NPE4	−72.9	−65.0	17.9	−99.0	3.9
NPE5	−74.5	−61.0	16.2	−97.7	3.2

Note. *T*_01_ is the onset of the relaxation transition; *T*_g1_ is the temperature of the first relaxation transition (relaxation transition of the crosslinked polymer matrix); Δ*T*_1_ is the range of the first relaxation transition; *T*_g2_ is the glass transition temperature of the second relaxation transition; Δ*T*_2_ is the range of the second relaxation transition.

**Table 3:** Self-diffusion coefficients and activation energy of diffusion on ^1^H nucleus.

*D_s_*, m^2^ s^−1^			E_a_, kJ mol^−1^	
	EC	EMIBF_4_	EC	EMIBF_4_
NPE1	3.99 × 10^−12^	—	42.5	—
NPE2	8.52 × 10^−12^	4.33 × 10^−12^	36.1	37.2
NPE3	1.70 × 10^−11^	9.99 × 10^−12^	30.0	29.9
NPE4	2.62 × 10^−11^	1.62 × 10^−11^	31.6	30.8
NPE5	2.70 × 10^−11^	1.70 × 10^−11^	29.2	28.3
EMIBF_4_	—	5.15 × 10^−11^	—	20.9

**Table 4:** Self-diffusion coefficients and activation energy of diffusion on ^7^Li and ^19^F nuclei.

*D_s_*, m^2^ s^−1^			E_a_, kJ mol^−1^	
	^7^Li	^19^F (LiBF_4_ + EMIBF_4_)	^7^Li	^19^F (LiBF_4_ + EMIBF_4_)
NPE1	1.15 × 10^−13^	1.42 × 10^−12^	51.6	37.5
NPE2	3.19 × 10^−13^	3.41 × 10^−12^	44.5	33.2
NPE3	7.26 × 10^−13^	6.85 × 10^−12^	42.6	29.1
NPE4	1.47 × 10^−12^	1.24 × 10^−11^	37.1	27.1
NPE5	1.90 × 10^−12^	1.30 × 10^−11^	35.8	27.6
EMIBF_4_	—	3.90 × 10^−11^	—	27.0

**Table 5:** Conductivity of the NPEs and their effective activation energy.

No.	σ, mS cm^–1^ (25 °C)	*E_a_*, kJ mol^−1^	
		−40 ÷ 15 °C	25 ÷ 100 °C
NPE1	0.03	37.8 ± 2.4	31.1 ± 1.6
NPE2	0.8	38.0 ± 0.3	24.5 ± 2.0
NPE3	1.8	33.2 ± 1.6	18.6 ± 0.3
NPE4	5.0	24.1 ± 1.1	14.0 ± 1.4
NPE5	6.2	23.5 ± 0.9	13.7 ± 1.4

## Data Availability

Not applicable.

## Supplementary Materials

The following supporting information can be downloaded at https://www.mdpi.com/article/10.3390/membranes13060548/s1. Table of Contents; Table S1: Compositions of the nanocomposite polymer gel electrolytes; Synthesis of the nanocomposite polymer electrolytes, Figure S1: Calculated structure of (a) the initial PEGDA, consisting of 6 (—CH2CH2O—) units; (b) the simplest element of a polymer network, consisting of 4 connected PEGDA fragments, where the dotted line indicates the places of their crosslinking, in which broken C—C bonds are replaced by C—H bonds; and (c) the theoretical IR spectra of these models, Figure S2: The calculated structure of (a) the solvate complex of the Li+ cation with three EC molecules and the BF4— counterion; and (b) the theoretical IR spectrum of this solvate complex, Figure S3: FTIR spectra of NPE4 and NPE5 versus EC solvent, PEGDA polymer and the polymer electrolyte without SiO2 and EMIBF4 in a range of 1650÷1900 cm−1, Figure S4: FTIR spectra of (a) NPE1, (b) NPE2, (c) NPE3 and (d) NPE4 before and after TGA experiments up to 150 °C, Figure S5: 7Li NMR spectra of the polymer electrolytes (a) NPE1, (b) NPE2, (c) NPE3, (d) NPE4, (e) NPE5 and (f) EMIBF4, Figure S6: 11B NMR spectra of the polymer electrolytes (a) NPE1, (b) NPE2, (c) NPE3, (d) NPE4, (e) NPE5 and (f) EMIBF4, Figure S7: 19F NMR spectra of the polymer electrolytes (a) NPE1, (b) NPE2, (c) NPE3, (d) NPE4, (e) NPE5 and (f) EMIBF4, Figure S8: The 13C—1H HSQC spectrum of the polymer electrolyte NPE1, Figure S9: Diffusion decays of NPE1-5 on (a) 7Li and (b) 19F; 1H nuclei for (c) EMIBF4 and (d) EC, Figure S10: Nyquist plots of the SS/NPE/SS cell at room temperature (a) and equivalent scheme (b), where R1 is the resistance of the electrolyte, and R2 is the resistance at the SiO2/electrolyte interface; CPE1 is the capacity of electrical double layer, Table S2: Conductivity of the nanocomposite polymer gel electrolytes, Figure S11: (a) Nyquist plots of the Li/NPE4/Li cells after assembly; (b) the equivalent scheme, where R1 is the electrolyte resistance, and R2 is the resistance of the Li/NPE4 interface; CPE1 is the capacity of electrical double layer; and (c) the visualization of the equivalent circuit parameter calculation performed with the program ZView2, Figure S12: Dependence of the Coulomb efficiency on the cycle number for the Li/NPE3/LiOTAP cells at the C/2 current rate in a voltage range of 0.7–3.5 V, Figure S13: FTIR spectra of the polymer electrolytes NPE3 and NPE3 before and after cycling cells versus LiOTAP, Figure S14: (a) The charge–discharge profiles and (b) dependence of the discharge capacity on the cycle number for the Li/EMIBF4/LiOTAP cells at the C/10 current rate in a voltage range of 0.7–3.5 V, Figure S15: The models of di-radical from imidazolium cation (a) and the adsorption of EMI•—radical on the surface of SiO2 nanoparticle (b), Figure S16: The theoretical IR spectra of dimer EMIBF4 and di-radical EMI (a); the experimental spectra of NPE3 sample before and after cell cycling (b), Table S3: Attachment energy of various ionic complexes to the surface of a SiO_2_ nanoparticle. References [45,46] are cited in there.
