# Supplementary material for: New Network Polymer Electrolytes Based on Ionic Liquid and SiO2 Nanoparticles for Energy Storage Systems
Source: Membranes (Basel). 2023 May 24;13(6):548. doi: 10.3390/membranes13060548 (PMC10301224; doi:10.3390/membranes13060548)
Supplement: Supplementary file 1 [file membranes-13-00548-s001.zip › membranes-2391654-supplementary.pdf]

Electronic supplementary information

New Network Polymer electrolytes based on Ionic liquid and SiO_2 nanoparticles for Energy storage systems

Kyunsylu G. Khatmullina, Nikita A. Slesarenko, Alexander V. Chernyak, Guzaliya R. Baymuratova, Alena V. Yudina, Mikhail P. Berezin, Galiya Z. Tulibaeva, Anna A. Slesarenko, Alexander F. Shestakov and Olga V. Yarmolenko

Table of Contents

Table S1. Compositions of the nanocomposite polymer gel electrolytes 3

Synthesis of the Nanocomposite Polymer Electrolyte 3

Figure S1. Calculated structure of **(a)** the initial PEGDA, consisting of 6 (—CH_2CH_2O—) units; **(b)** the simplest element of a polymer network, consisting of 4 connected PEGDA fragments, where the dotted line indicates the places of their crosslinking, in which broken C—C bonds are replaced by C—H bonds; and **(c)** the theoretical IR spectra of these models..................................4

Figure S2. The calculated structure of **(a)** the solvate complex of the Li^+ cation with three EC molecules and the BF_4^- counterion; and **(b)** the theoretical IR spectrum of this solvate complex.5

Figure S3. FTIR spectra of NPE4 and NPE5 versus EC solvent, PEGDA polymer, and the polymer electrolyte without SiO_2 and $EMIBF_4$ in a range of 1650÷1900 cm^{-1} 6

Figure S4. FTIR spectra of **(a)** NPE1; **(b)** NPE2; **(c)** NPE3, and **(d)** NPE4 before and after TGA experiments up to 150 °C 8

Figure S5. 7Li NMR spectra of the polymer electrolytes **(a)** NPE1; **(b)** NPE2; **(c)** NPE3; **(d)** NPE4; **(e)** NPE5, and **(f)** $EMIBF_4$...9

Figure S6. 11B NMR spectra of the polymer electrolytes **(a)** NPE1; **(b)** NPE2; **(c)** NPE3; **(d)** NPE4; **(e)** NPE5, and **(f)** $EMIBF_4$ 10

Figure S7. 19F NMR spectra of the polymer electrolytes **(a)** NPE1; **(b)** NPE2; **(c)** NPE3; **(d)** NPE4; **(e)** NPE5, and **(f)** $EMIBF_4$ 11

Figure S8. The 13C—1H HSQC spectrum of the polymer electrolyte NPE1.................................12

Figure S9. Diffusion decays of NPE1-5 on **(a)** 7Li; **(b)** 19F; 1H nuclei for **(c)** $EMIBF_4$ and **(d)** EC 13

Figure S10. Nyquist plots of the SS/NPE/SS cell at room temperature **(a)** and equivalent scheme **(b)**, where R1 is the resistance of the electrolyte, and R2 is the resistance at the SiO_2/electrolyte interface; CPE1 is the capacity of electrical double layer 14

Table S2. Conductivity of the nanocomposite polymer gel electrolytes 15

Figure S11. (a) Nyquist plots of the Li/NPE4/Li cells after assembly; (b) the equivalent scheme, where R1 is the electrolyte resistance, and R2 is the resistance of the Li/NPE4 interface, CPE1 is the capacity of electrical double layer; and (c) the visualization of the equivalent circuit parameters calculation performed by the program ZView2..16

Figure S12. Dependence of the Coulomb efficiency on the cycle number for the Li/NPE3/LiOTAP cells at the C/2 current rate in a voltage range of 0.7–3.5 V................................17

Figure S13. FTIR spectra of the polymer electrolytes NPE3 and NPE3 before and after cycling cells versus LiOTAP..18

Figure S14. (a) The charge-discharge profiles and **(b)** dependence of the discharge capacity on the cycle number for the Li/$EMIBF_4$/LiOTAP cells at the C/10 current rate in a voltage range of 0.7–3.5 V...19

Figure S15. The models of diradical from imidazolium cation **(a)** and the adsorption of $EMI^{\bullet}$ — radical on the surface of SiO_2 nanoparticle **(b)** 20

Figure S16. The theoretical IR spectra of dimer $EMIBF_4$ and di-radical EMI (a), the experimental spectra of NPE3 sample before and after cell cycling (b) 21

Table S3. Attachment energy of various ionic complexes to the surface of a SiO_2 nanoparticle .22

Table S1. Compositions of the nanocomposite polymer gel electrolytes

No.	PEGDA, wt.%	$LiBF_4$, wt.%	$EMIBF_4$, wt.%	EC, wt.%	SiO_2, wt.%	PB, wt.%
NPE0	31	4	52	12	0	1
NPE1	64.2	8.6	0	24.2	2	1
NPE2	46.7	6.3	26.4	17.6	2	1
NPE3	37.6	5	40.2	14.2	2	1
NPE4	30.2	4.1	51.3	11.4	2	1
NPE5	29.0	4	49.0	11.0	6	1

Synthesis of the Nanocomposite Polymer Electrolyte

Synthesis of samples of the polymer electrolyte was carried out by radical polymerization in the presence of 1 wt.% BP. After mixing the initial components, the solution was poured to a glass reactor treated with an adhesive with a Teflon spacer with a thickness of 0.2—0.3 mm. The synthesis procedure produced transparent films with 0.2—0.3 mm thick determined by the thickness of the Teflon spacer.

The NPE films were obtained after thermal solidification according to the following regime: 60 °C for 3 h, 70 °C for 1 h, 80 °C for 1 h. This regime for compositions with a large amount of ionic liquid (6 mol) was used. The ionic liquid accelerates the polymerization reaction, and this regime is sufficient for complete cross-linking of C=C bonds [45].

The thermal solidification at 100°C for 1 h for NPE with $EMIBF_4$ (0, 2, and 4 mol) was used additionally. This regime was ensured complete crosslinking of C=C bonds, because the SiO_2 nanoparticles hinder the PEGDA polymerization reaction [46].

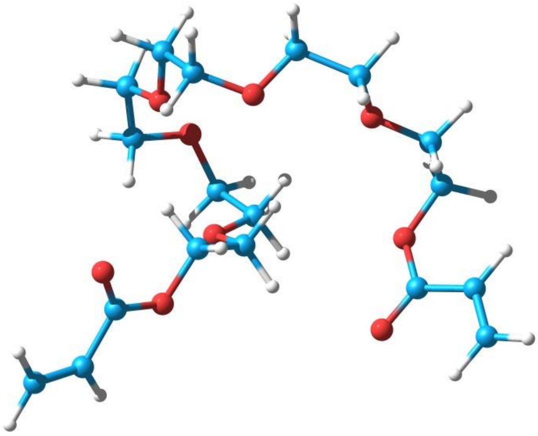

(a)

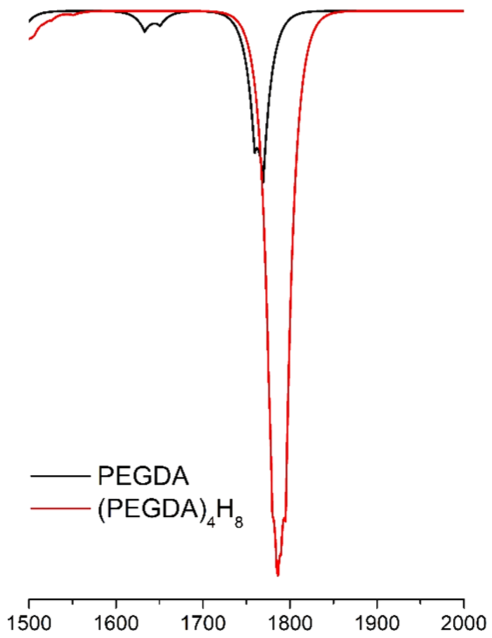

(c)

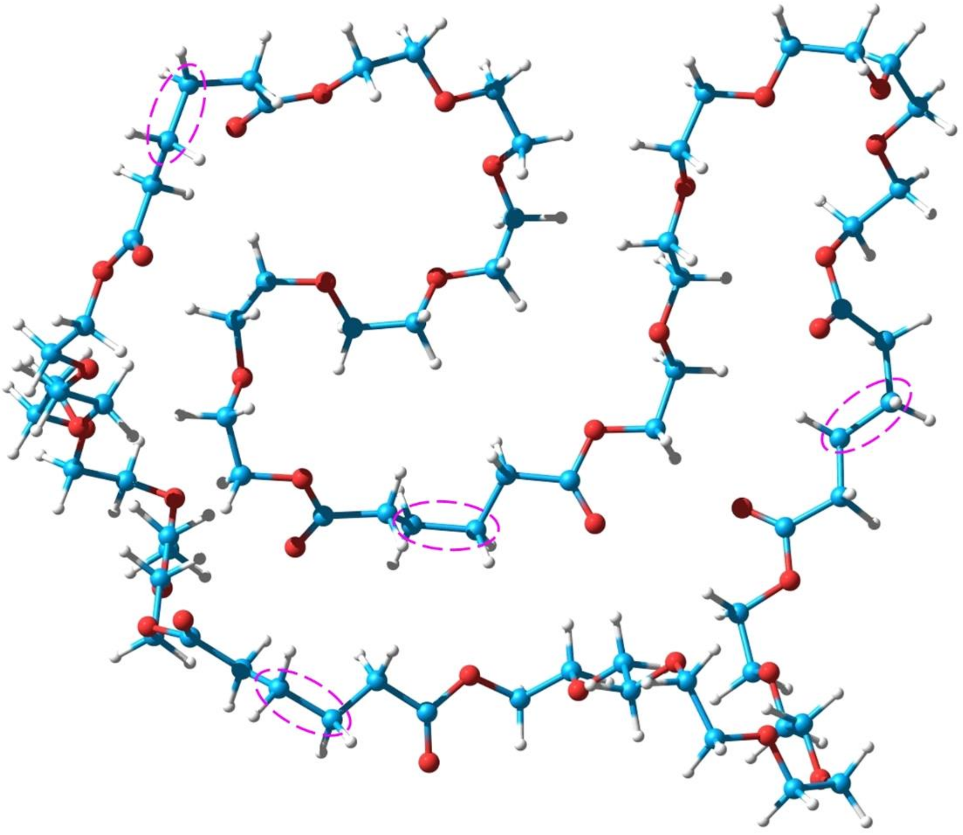

(b)

Figure S1. Calculated structure of **(a)** the initial PEGDA, consisting of 6 (—CH_2CH_2O—) units; **(b)** the simplest element of a polymer network, consisting of 4 connected PEGDA fragments, where the dotted line indicates the places of their crosslinking, in which broken C—C bonds are replaced by C—H bonds; and **(c)** the theoretical IR spectra of these models

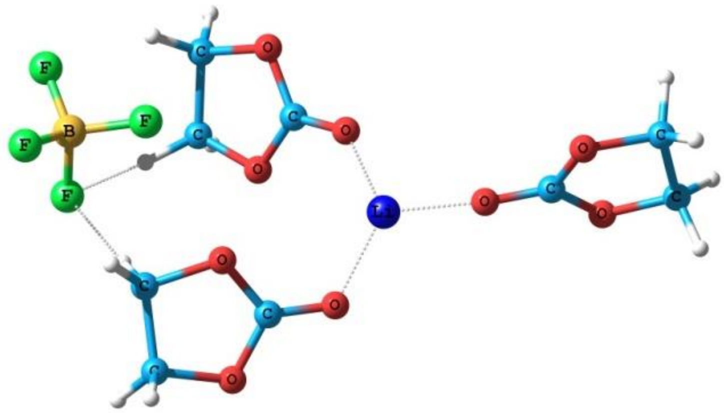

(a)

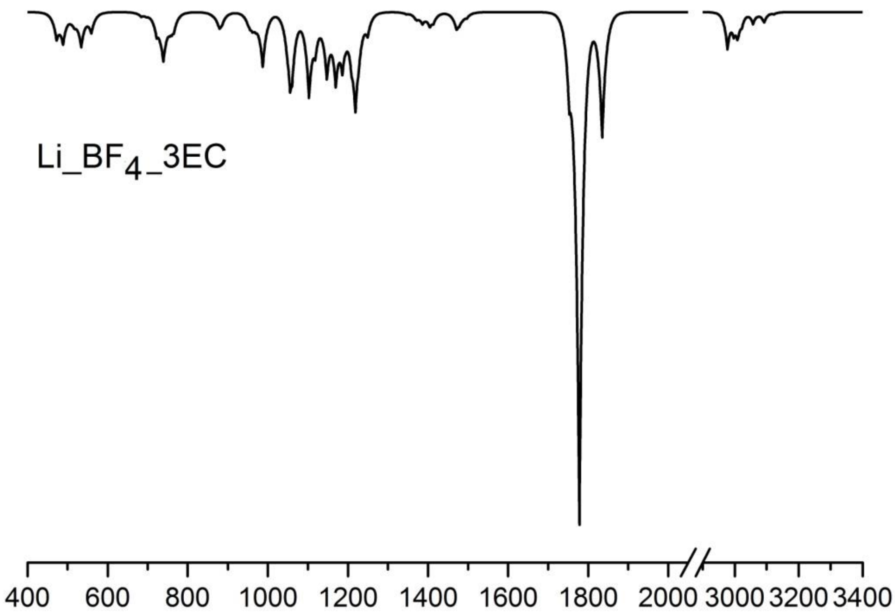

(b)

Figure S2. The calculated structure of **(a)** the solvate complex of the Li^+ cation with three EC molecules and the BF_4^- counterion; and **(b)** the theoretical IR spectrum of this solvate complex

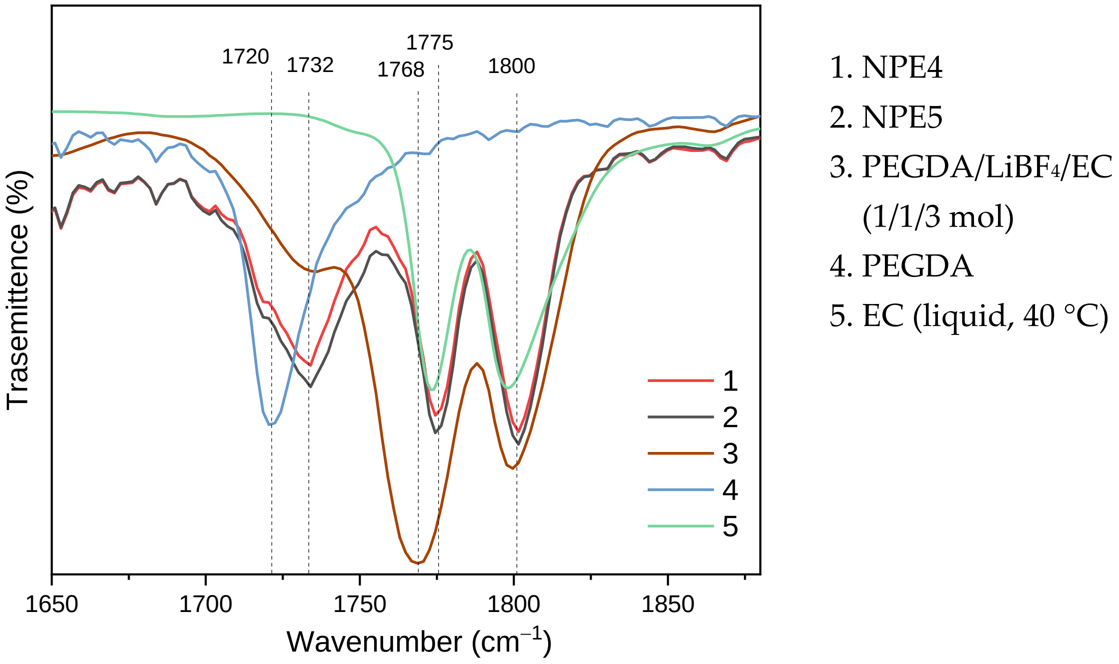

Figure S3. FTIR spectra of NPE4 and NPE5 versus EC solvent, PEGDA polymer, and the polymer electrolyte without SiO_2 and $EMIBF_4$ in a range of 1650÷1900 cm^{-1}

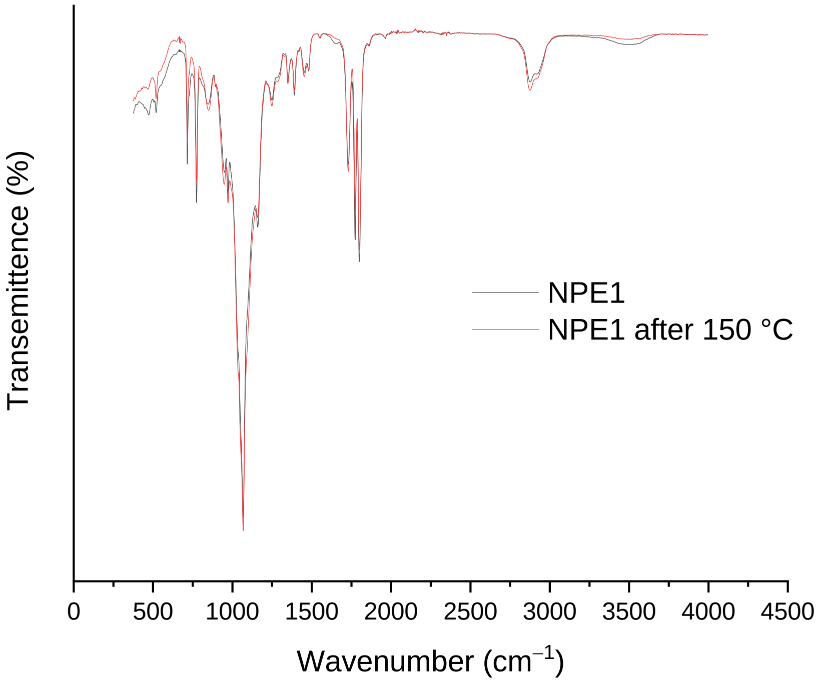
Transemittence (%)
0
500
1000
1500
2000
2500
3000
3500
4000
4500
Wavenumber (cm−1)
NPE1
NPE1 after 150 °C

(a)

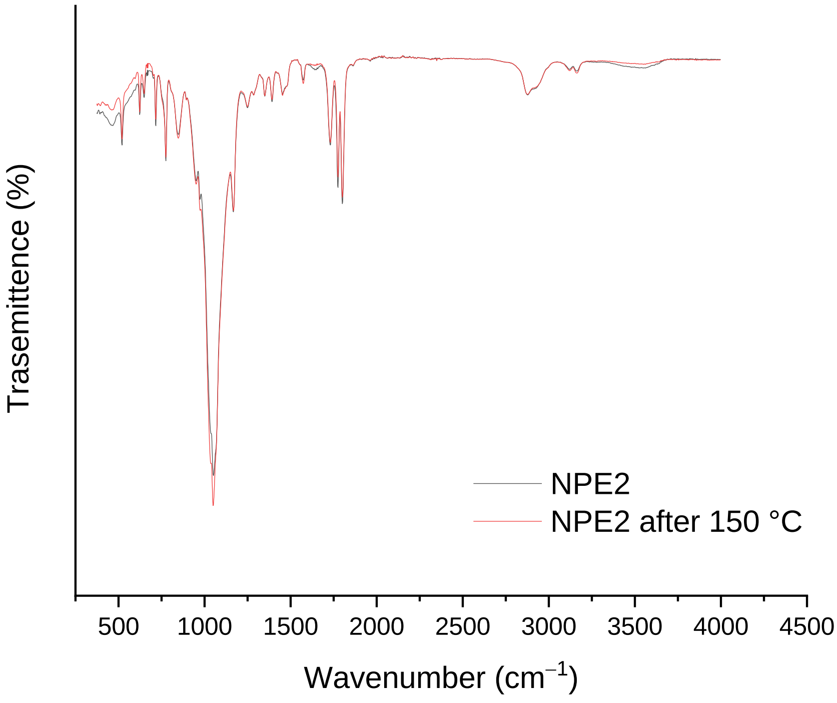
Trasemittence (%)
500
1000
1500
2000
2500
3000
3500
4000
4500
Wavenumber (cm−1)
NPE2
NPE2 after 150 °C

(b)

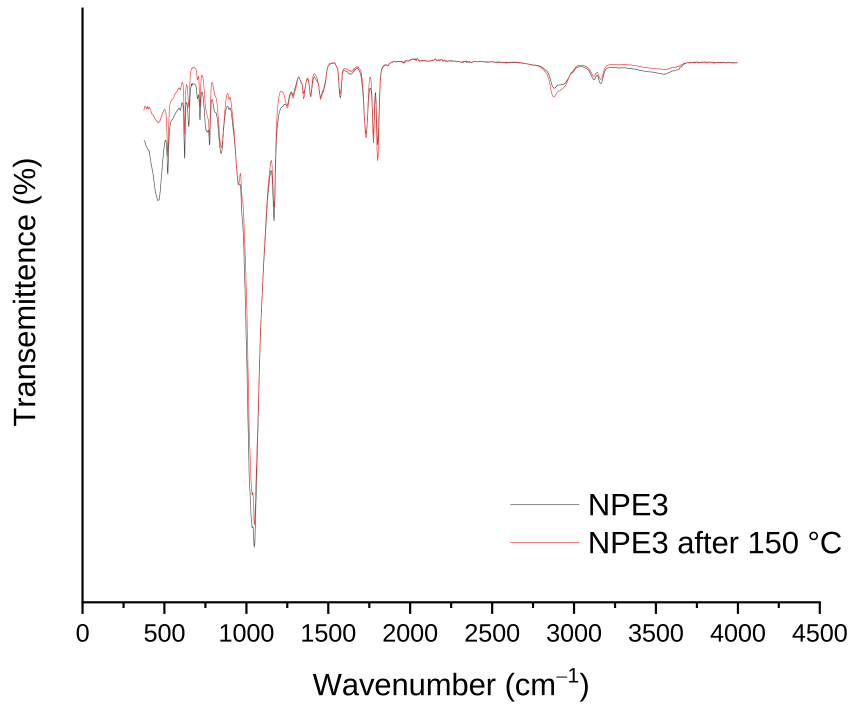

(c)

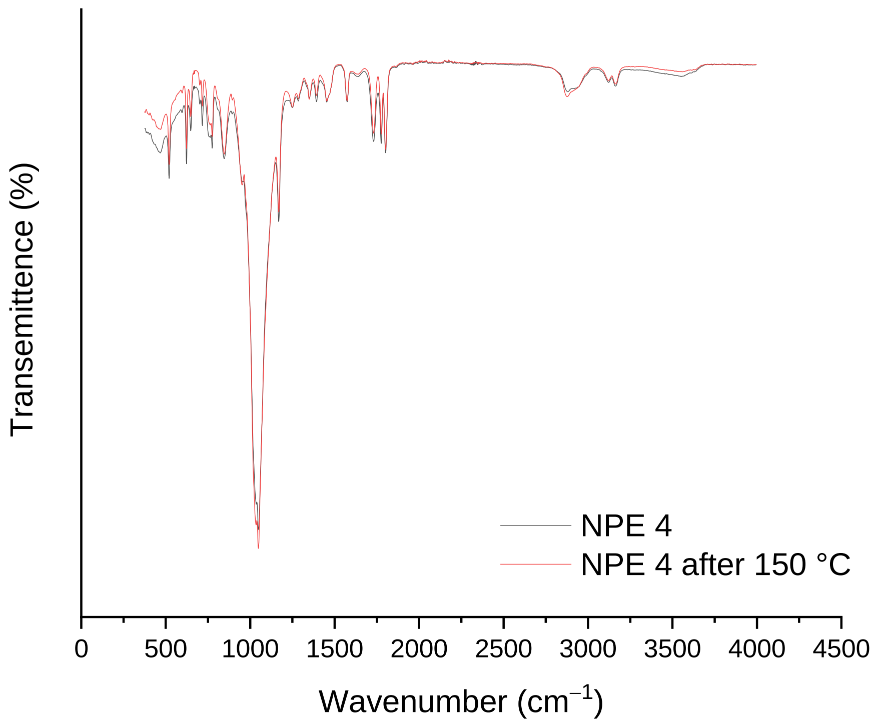

(d)

Figure S4. FTIR spectra of **(a)** NPE1; **(b)** NPE2; **(c)** NPE3, and **(d)** NPE4 before and after TGA experiments up to 150 °C

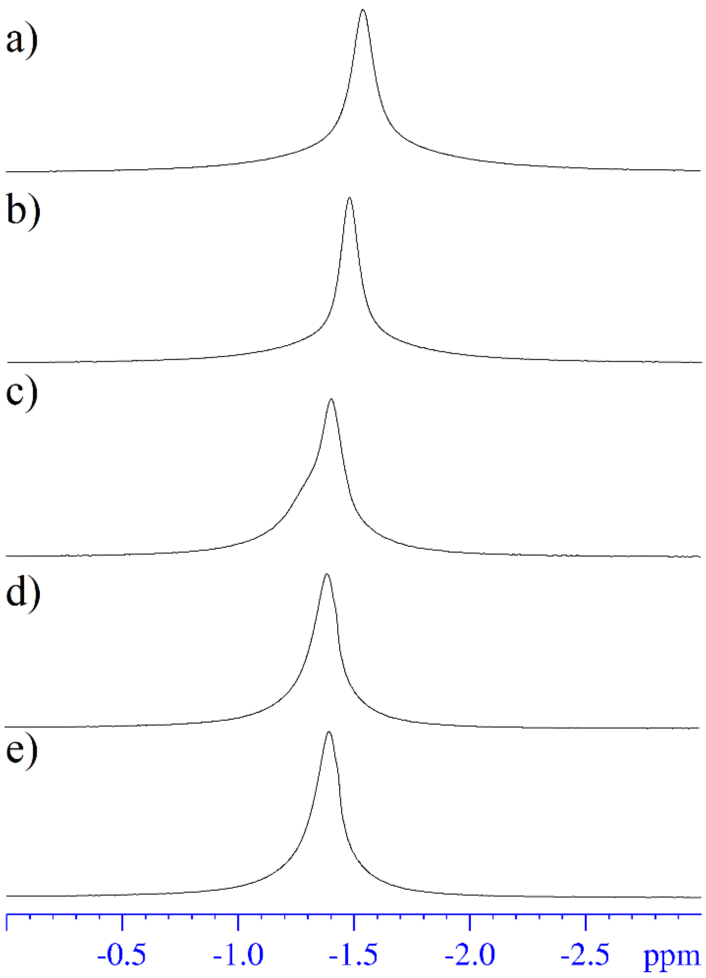

Figure S5. 7Li NMR spectra of the polymer electrolytes **(a)** NPE1; **(b)** NPE2; **(c)** NPE3; (**d**) NPE4; **(e)** NPE5

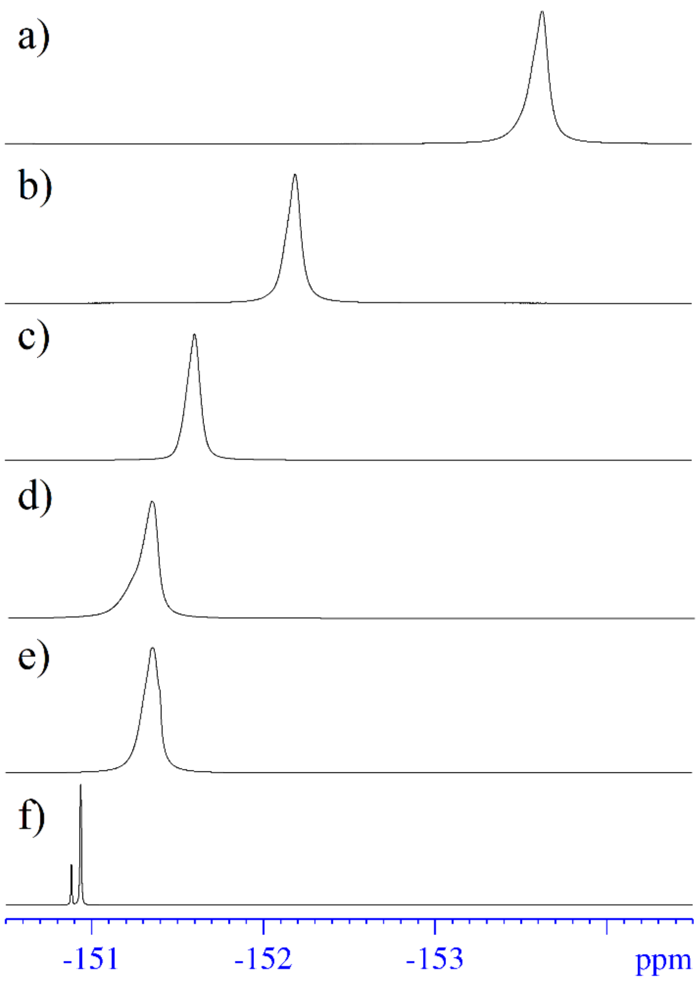

Figure S6. 11B NMR spectra of the polymer electrolytes **(a)** NPE1; **(b)** NPE2; **(c)** NPE3; **(d)** NPE4; **(e)** NPE5, and **(f)** EMIBF$_4$

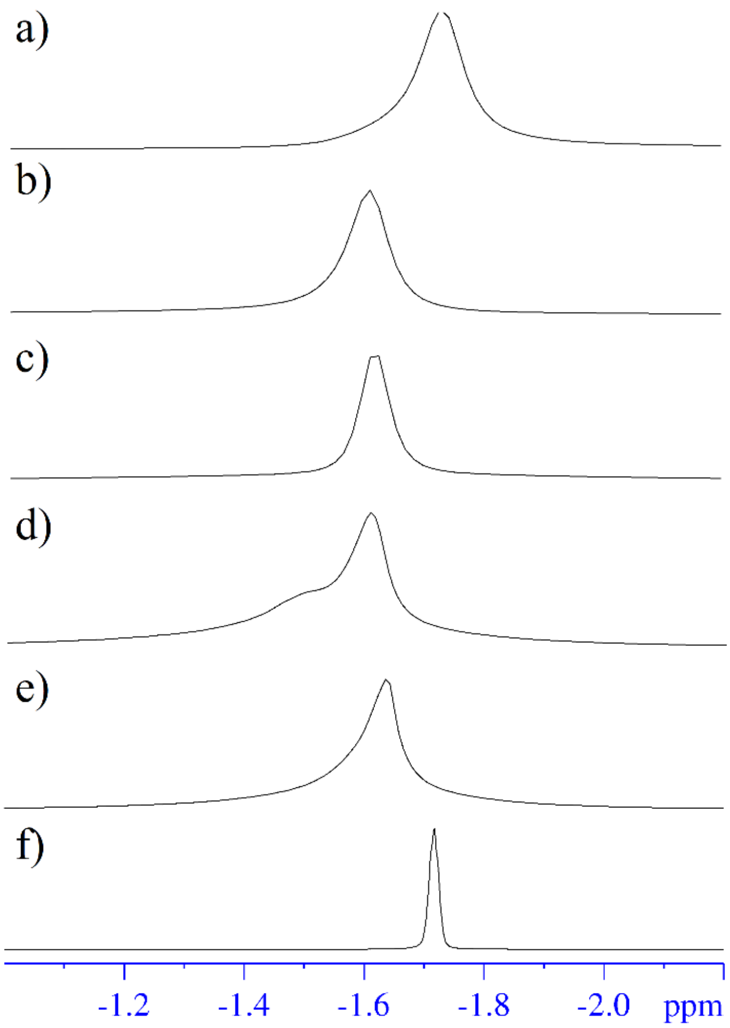

Figure S7. 19F NMR spectra of the polymer electrolytes **(a)** NPE1; **(b)** NPE2; **(c)** NPE3; **(d)** NPE4; **(e)** NPE5, and **(f)** $EMIBF_4$

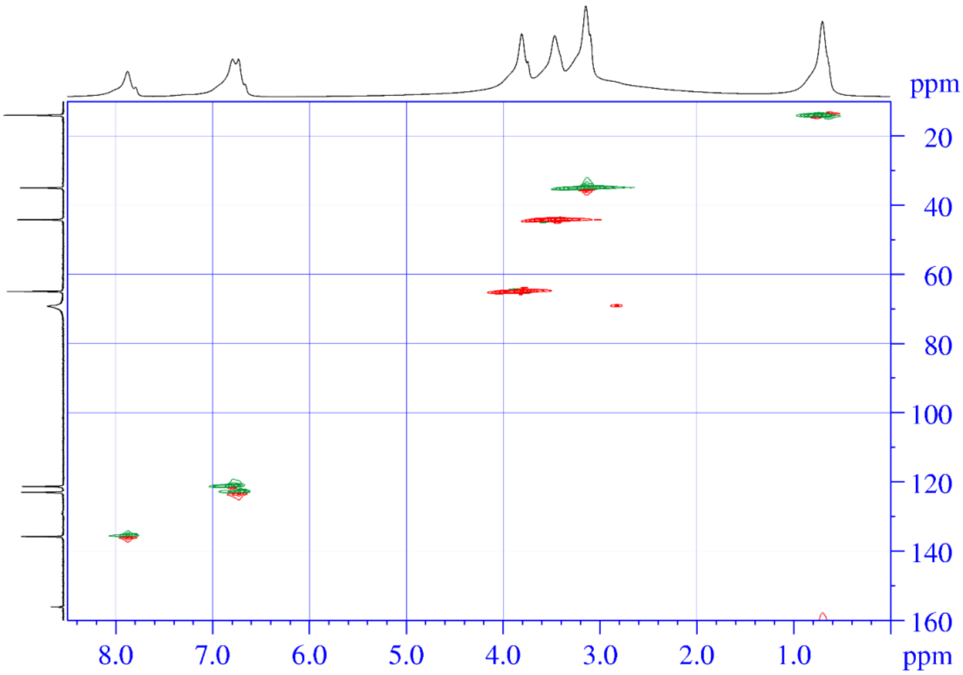

Figure S8. The 13C—1H HSQC spectrum of the polymer electrolyte NPE1

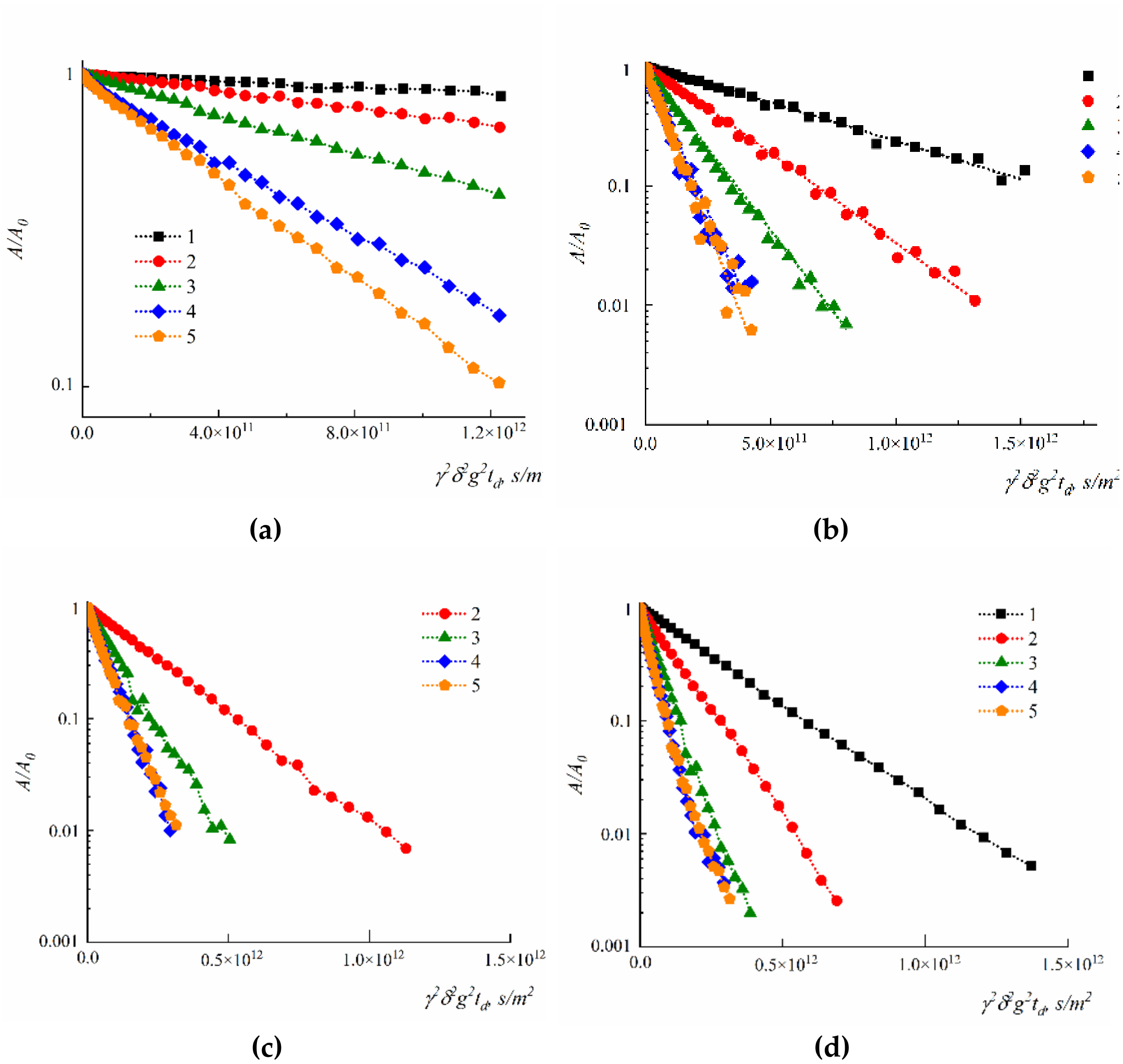

Figure S9. Diffusion decays of NPE1-5 on **(a)** 7Li; **(b)** 19F; 1H nuclei for **(c)** EMIBF$_4$ and **(d)** EC

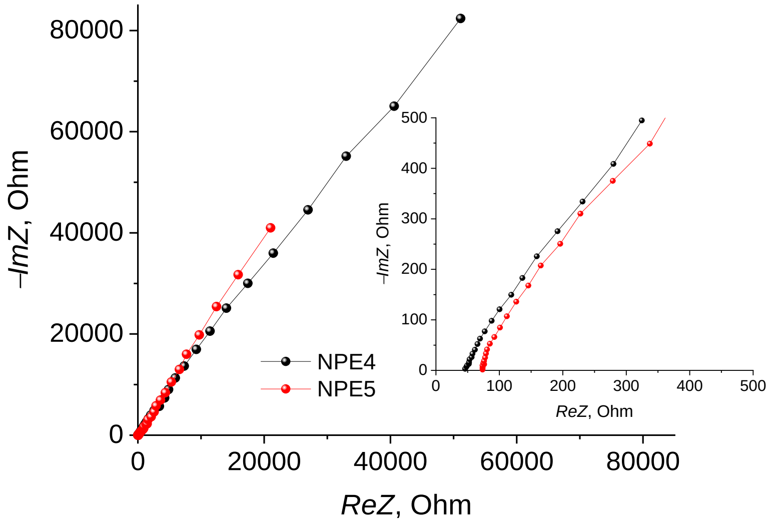

(a)

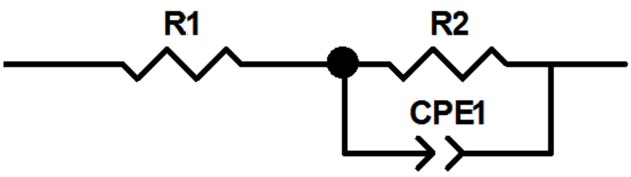

(b)

Figure S10. Nyquist plots of the SS/NPE/SS cell at room temperature **(a)** and equivalent scheme **(b)**, where R1 is the resistance of the electrolyte, and R2 is the resistance at the SiO_2/electrolyte interface; CPE1 is the capacity of electrical double layer

Table S2. Conductivity of the nanocomposite polymer gel electrolytes

T, °C	Conductivity, mS cm^{-1}				
	NPE1	NPE2	NPE3	NPE4	NPE5
−40	0.0004	0.01	0.04	0.3	0.5
−21	0.0016	0.05	0.2	0.8	1.3
−15	0.0019	0.08	0.3	1.2	1.8
0	0.007	0.2	0.6	2.2	3.4
15	0.02	0.5	1.1	3.3	5.0
25	0.03	0.8	1.8	5.0	6.2
40	0.05	1.4	2.7	7.8	9.6
60	0.13	3.0	4.1	10.6	13.4
80	0.20	4.5	5.8	13.6	16.2
100	0.34	5.8	8.2	15.8	19.8

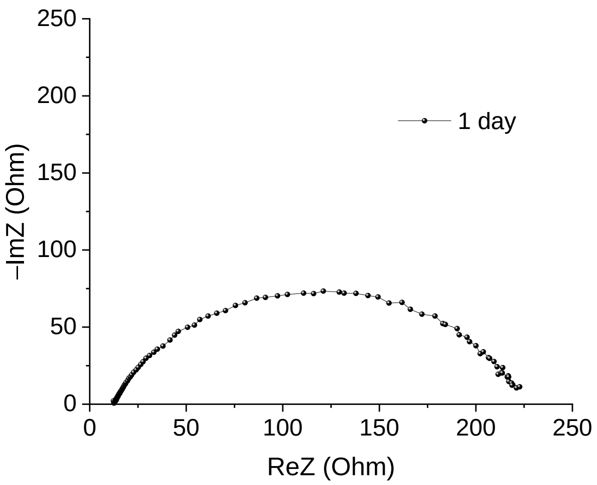

(**a**)

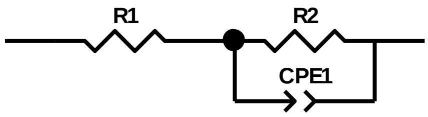

(**b**)

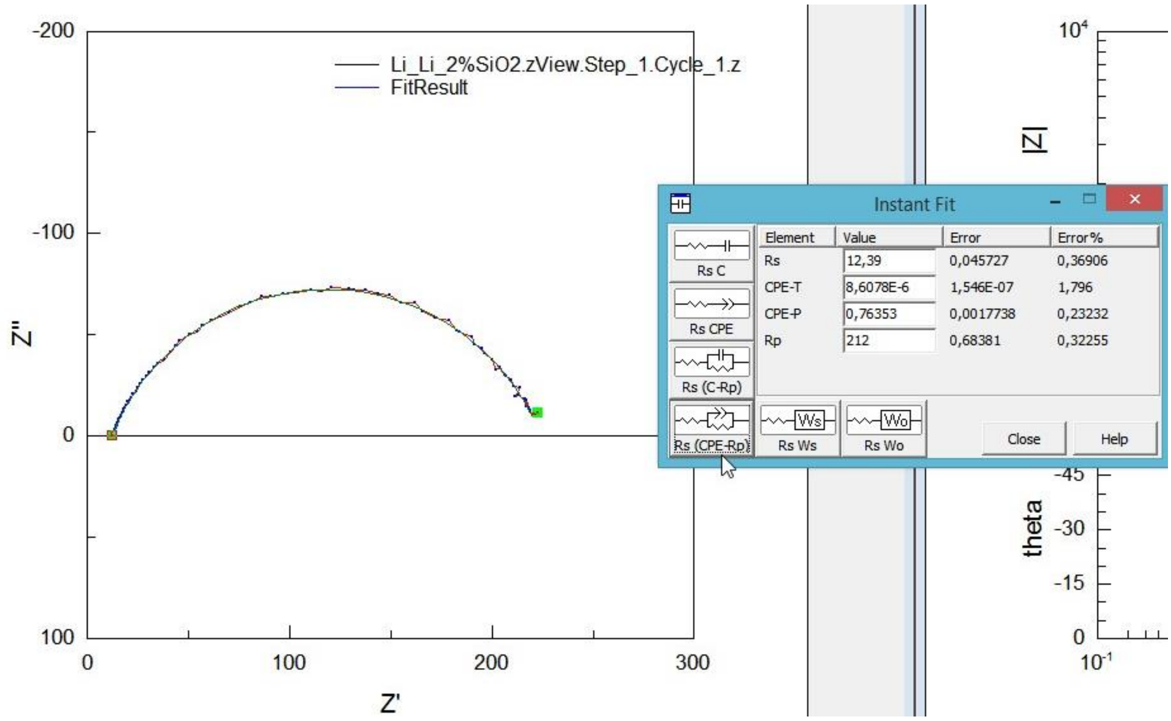

(**c**)

Figure S11. (a) Nyquist plots of the Li/NPE4/Li cells after assembly; (b) the equivalent scheme, where R1 is the electrolyte resistance, and R2 is the resistance of the Li/NPE4 interface, CPE1 is the capacity of electrical double layer; and (c) the visualization of the equivalent circuit parameters calculation performed by the program ZView2

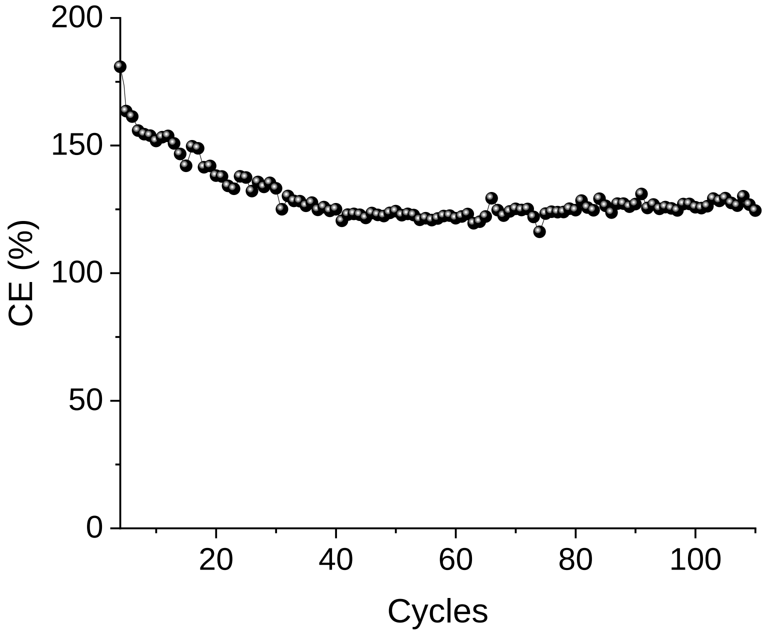

Figure S12. Dependence of the Coulomb efficiency on the cycle number for the Li/NPE3/LiOTAP cells at the C/2 current rate in a voltage range of 0.7–3.5 V.

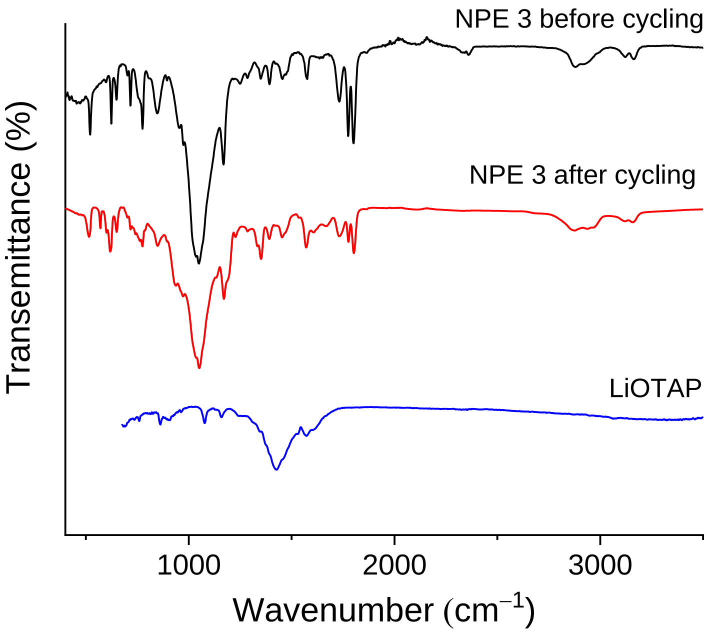

Figure S13. FTIR spectra of the polymer electrolytes NPE3 and NPE3 before and after cycling cells versus LiOTAP

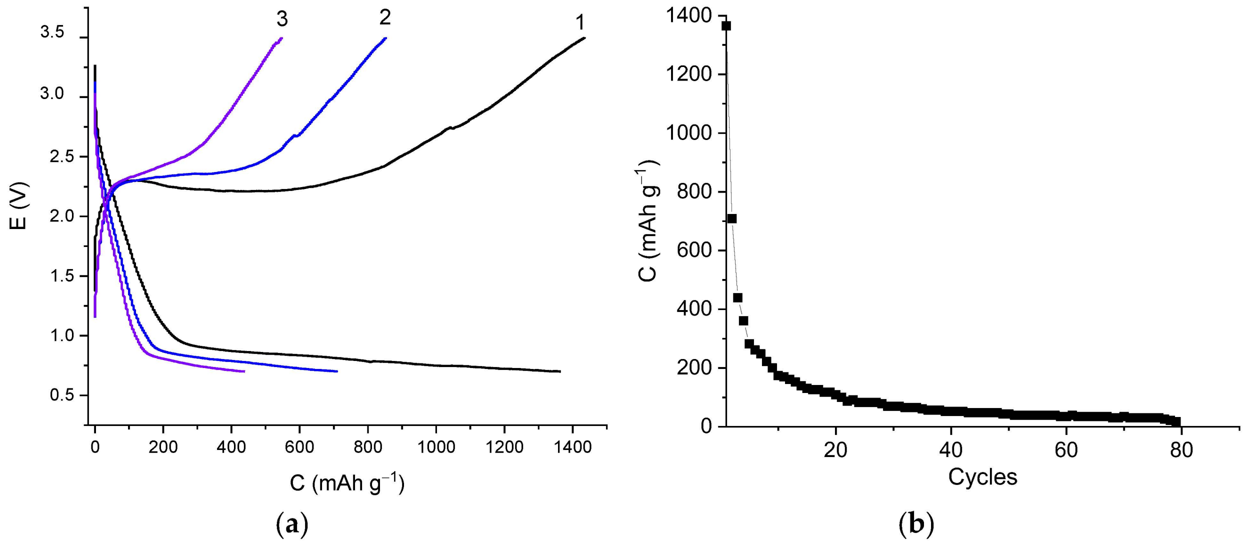

Figure S14. (a) The charge-discharge profiles and **(b)** dependence of the discharge capacity on the cycle number for the Li/EMIBF$_4$/LiOTAP cells at the C/10 current rate in a voltage range of 0.7–3.5 V

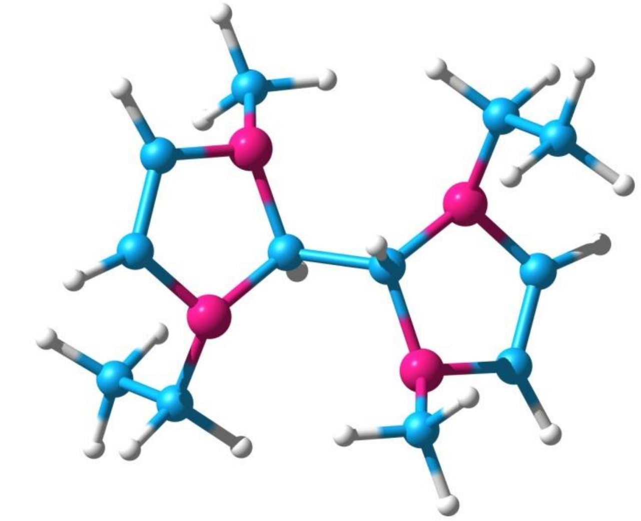

(a)

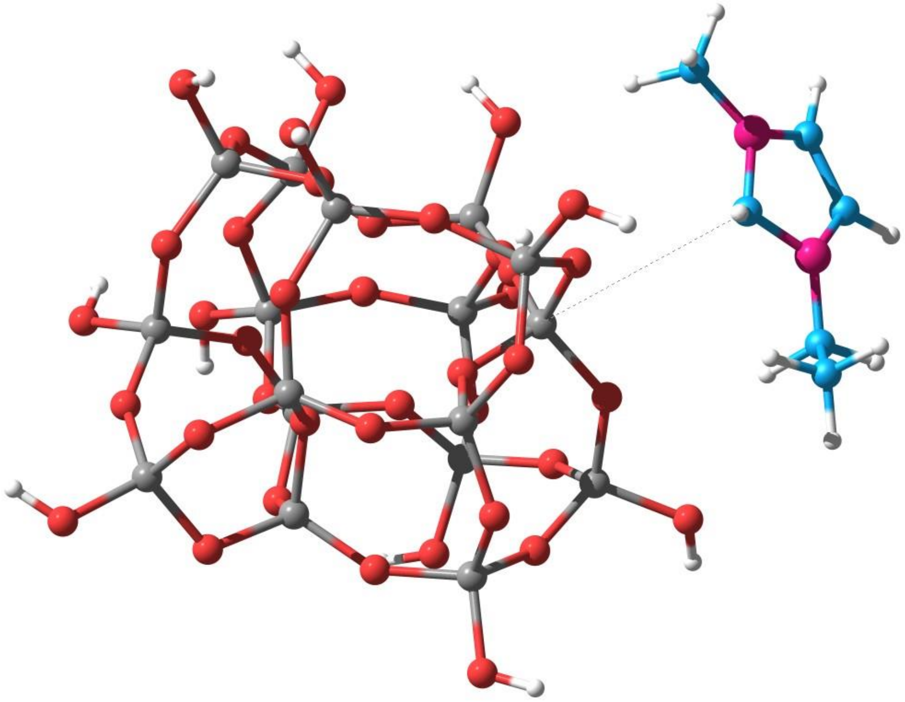

(b)

Figure S15. The models of diradical from imidazolium cation **(a)** and the adsorption of EMI$^{\bullet}$ — radical on the surface of SiO_2 nanoparticle **(b)**

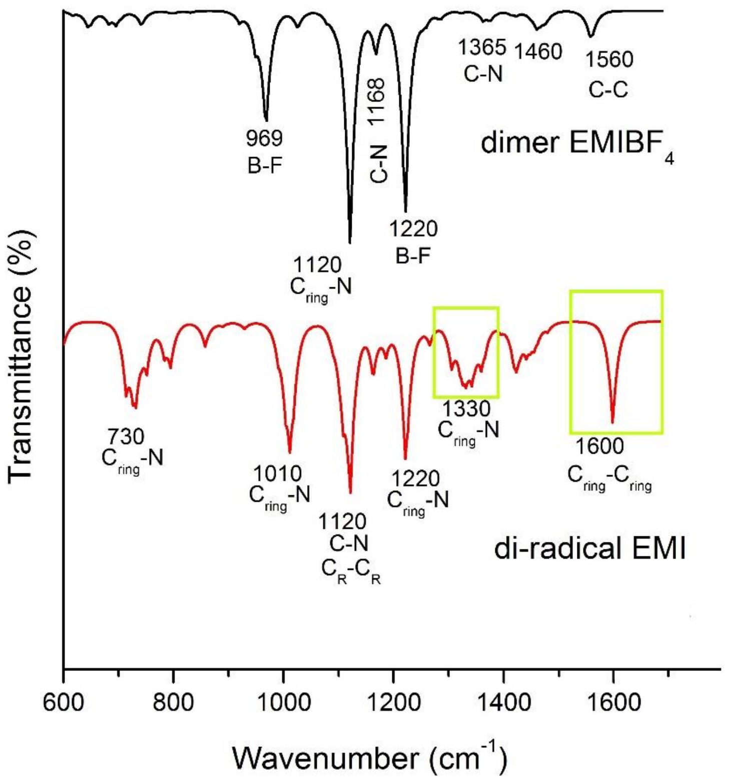

(a)

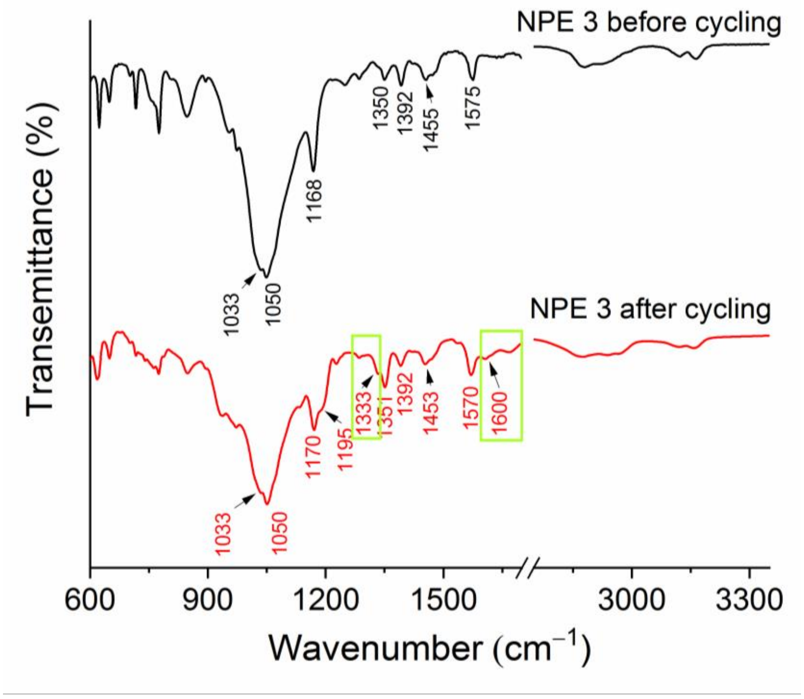

(b)

Figure S16. The theoretical IR spectra of dimer $EMIBF_4$ and di-radical EMI (a), the experimental spectra of NPE3 sample before and after cell cycling (b)

Table S3. Attachment energy of various ionic complexes to the surface of a SiO_2 nanoparticle

	**Energy change**	
	**Kcal mol⁻¹**	**relatively**
**Model I**	−23.5	$Si_{17}O_{28}(OH)_{12}$ + $(LiBF_4)_2$
**Model II**	−33.6	$Si_{17}O_{28}(OH)_{12}$ + $(EMIBF_4)_2$
**Model IIIa**	−58.1	$Si_{17}O_{28}(OH)_{12}$ + $(LiBF_4)_2$ + $(EMIBF_4)_2$
	−34.6	$Si_{17}O_{28}(OH)_{12}(LiBF_4)_2$ + $(EMIBF_4)_2$
**Model IIIb**	−74.6	$Si_{17}O_{28}(OH)_{12}$ + $(LiBF_4)_2$ + $(EMIBF_4)_2$
	−51.1	$Si_{17}O_{28}(OH)_{12}(LiBF_4)_2$ + $(EMIBF_4)_2$
